# Pseudomonas aeruginosa Initiates a Rapid and Specific Transcriptional Response during Surface Attachment

**DOI:** 10.1128/jb.00086-22

**Published:** 2022-04-25

**Authors:** Christopher J. Jones, Nikolas Grotewold, Daniel J. Wozniak, Erin S. Gloag

**Affiliations:** a Department of Microbial Infection and Immunity, Ohio State University, Columbus, Ohio, USA; b Department of Microbiology, Ohio State University, Columbus, Ohio, USA; Geisel School of Medicine at Dartmouth

**Keywords:** *Pseudomonas aeruginosa*, RNA-Seq, biofilm, surface adhesion, surface attachment

## Abstract

Chronic biofilm infections by Pseudomonas aeruginosa are a major contributor to the morbidity and mortality of patients. The formation of multicellular bacterial aggregates, called biofilms, is associated with increased resistance to antimicrobials and immune clearance and the persistence of infections. Biofilm formation is dependent on bacterial cell attachment to surfaces, and therefore, attachment plays a key role in chronic infections. We hypothesized that bacteria sense various surfaces and initiate a rapid, specific response to increase adhesion and establish biofilms. RNA sequencing (RNA-Seq) analysis identified transcriptional changes of adherent cells during initial attachment, identifying the bacterial response to an abiotic surface over a 1-h period. Subsequent screens investigating the most highly regulated genes in surface attachment identified 4 genes, *pfpI*, *phnA*, *leuD*, and *moaE*, all of which have roles in both metabolism and biofilm formation. In addition, the transcriptional responses to several different medically relevant abiotic surfaces were compared after initial attachment. Surprisingly, there was a specific transcriptional response to each surface, with very few genes being regulated in response to surfaces in general. We identified a set of 20 genes that were differentially expressed across all three surfaces, many of which have metabolic functions, including molybdopterin cofactor biosynthesis and nitrogen metabolism. This study has advanced the understanding of the kinetics and specificity of bacterial transcriptional responses to surfaces and suggests that metabolic cues are important signals during the transition from a planktonic to a biofilm lifestyle.

**IMPORTANCE** Bacterial biofilms are a significant concern in many aspects of life, including chronic infections of airways, wounds, and indwelling medical devices; biofouling of industrial surfaces relevant for food production and marine surfaces; and nosocomial infections. The effects of understanding surface adhesion could impact many areas of life. This study utilized emerging technology in a novel approach to address a key step in bacterial biofilm development. These findings have elucidated both conserved and surface-specific responses to several disease-relevant abiotic surfaces. Future work will expand on this report to identify mechanisms of biofilm initiation with the aim of identifying bacterial factors that could be targeted to prevent biofilms.

## INTRODUCTION

Biofilms formed by Pseudomonas aeruginosa play a key role in many chronic infections, including pulmonary infections of people with cystic fibrosis (CF), wounds, indwelling catheters, artificial joints, and ventilator-associated pneumonia (VAP) (1, 2). The prevalence of biofilm infections is very high in these situations, with biofilms being detected in up to 95% of VAP cases and 80% of adults with CF (1, 3).

Biofilms confer many advantages to bacterial populations, including resistance to desiccation (4), antimicrobial treatments (5, 6), immune cell mediators (7, 8), and phagocytosis (9–11). As a result, biofilms have been an important area of study for nearly 4 decades. Several groups have identified surface sensing and response mechanisms important for the initiation of biofilm formation, including type IV pilus-mediated regulation of cAMP (12, 13) and c-di-GMP regulation by the Wsp system (14). Previous studies that have determined transcriptional differences between planktonic and biofilm populations relied on mature biofilms, with early sampling beginning from 4 to 12 h postinitiation (15–19). This was largely due to technological hurdles, requiring a large population of cells necessary to generate sufficient RNA for transcriptional profiling.

While these studies have elucidated many important changes that occur as a biofilm matures, we were interested in the bacterial response during initial surface attachment. We hypothesized that bacteria sense surfaces and initiate a transcriptional response cascade early after attachment and that modulation of this transcriptional response would modify the ability of bacteria to bind and initiate biofilms. Here, we utilize an optimized RNA sequencing (RNA-Seq) procedure to generate transcriptional profiles from adherent populations both early after surface attachment (5 to 60 min) and upon attachment to different medically relevant abiotic surfaces. In both cases, we observed rapid shifts in genes implicated in metabolism, with these responses being largely surface specific.

Bacterial biofilms have been studied for decades; however, studies about the early events after surface attachment are an emerging field (19, 20). This study begins to address this gap by defining the kinetics of the P. aeruginosa transcriptional response to a surface as well as elucidating the specificity of these early transcriptional responses to various surfaces. We propose that these data will establish a framework for studies of early bacterial responses to surfaces as well as provide insights to guide targeted molecular studies of P. aeruginosa attachment.

## RESULTS AND DISCUSSION

### P. aeruginosa induces a rapid transcriptional response upon surface attachment.

**FIG 1:**
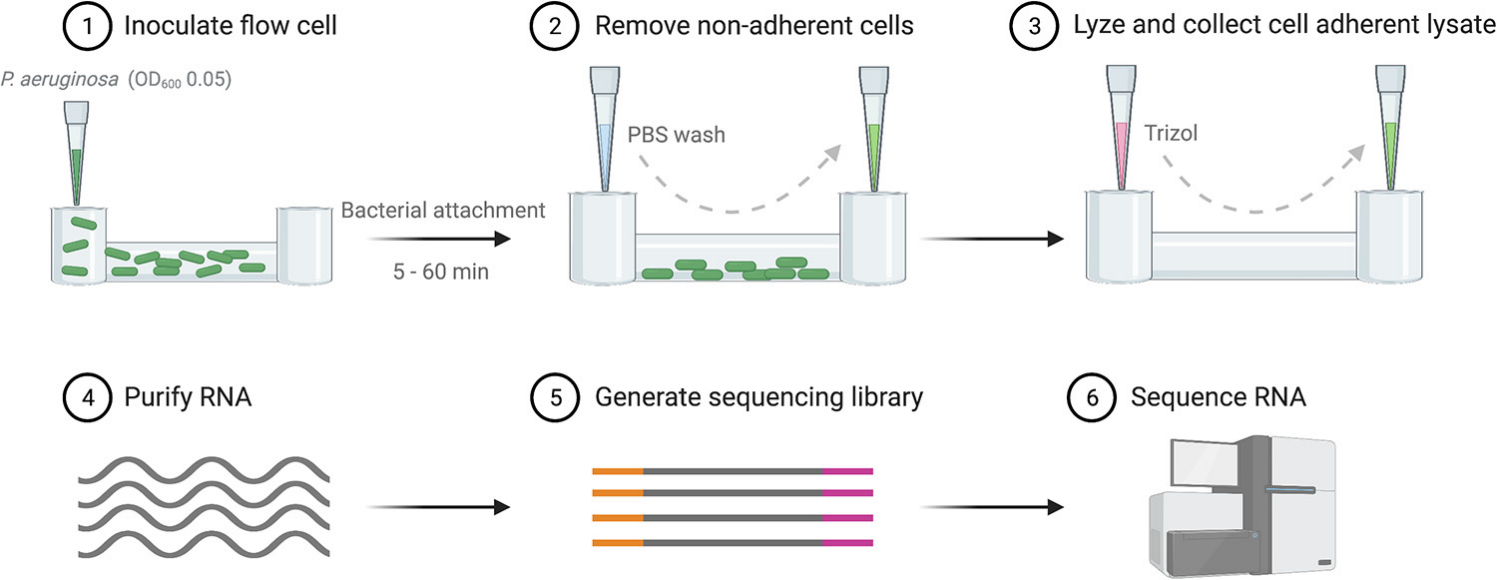
Schematic of the experimental design. Cells were allowed to adhere to a polycarbonate flow cell for the indicated times, and nonadherent cells were then removed via a PBS wash. Cells were lysed by the addition of TRIzol, and RNA was subsequently purified. A barcoded genomic sequencing library was generated and then sequenced to produce raw reads. The sequencing files were processed and aligned with Rockhopper. (Image created with BioRender.com.)

**FIG 2:**
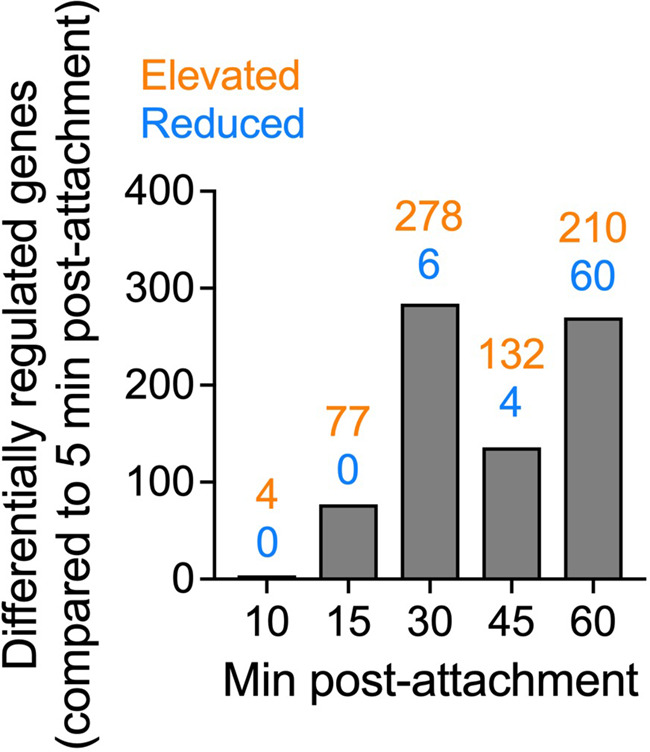
Gene regulation in response to surface exposure is rapid. Differential gene expression of adherent cell populations was determined between gene expression at the indicated time points and gene expression after 5 min of exposure to the surface (*q* ≤ 0.05). The number of differentially expressed genes at each time point is indicated.

Previous research investigating bacterial transcriptional changes during biofilm formation was limited to comparing planktonic cells to mature biofilms, starting at around 4 to 12 h postinoculation (15–19). We propose that the transcriptional response to surfaces may occur much earlier than what has traditionally been studied. To investigate the kinetics of bacterial responses to surfaces, we optimized a procedure to isolate RNA from adherent populations of P. aeruginosa strain PAO1 from 5 to 60 min postattachment on an Ibidi μ-Slide and generate libraries for sequencing (Fig. 1). Equivalent amounts of bacterial cells were observed attached to the surface, and equivalent amounts of RNA were isolated at each time point postattachment (see Fig. S1 in the supplemental material). Sequencing was performed on the Illumina HiSeq 4000 platform. Sequences were aligned to the PAO1 reference genome (21), and differentially expressed genes were determined using Rockhopper (22–24). Expression patterns at each time point were compared to the transcriptional profile at 5 min postattachment. This time point was chosen for comparison, rather than the planktonic population, to limit heterogeneity and reduce the number of genes associated with the planktonic lifestyle as confounding variables. The data concur with this assumption, as only 4 genes were differentially expressed when comparing the 5- and 10-min samples, while more responses were observed at later time points, with the transcriptional response beginning at 15 min postattachment and peaking at 30 min postattachment (Fig. 2 and Table 1). Gene expression analysis was sorted to include only significantly regulated genes (*q* < 0.05). The kinetics of gene regulation for each gene can be observed in Table S1. In total, 453 genes were differentially regulated between 5 min postattachment and at least one of the later time points. The expression of most of the regulated genes was elevated (390 genes) rather than reduced (63 genes) upon surface attachment (Fig. 2).

**TABLE 1:** Curated surface-regulated genes

PA no.	Gene name	Gene product	Fold change compared to 5 min^*a*^			
			15 min	30 min	45 min	60 min
PA0034		Two-component response regulator		2.08		
PA0155	*pcaR*	Transcriptional regulator		2.56		
PA0159		Transcriptional regulator	2.474	2	2.368	2.211
PA0177		Purine-binding chemotaxis protein		3.042		5.75
PA0178		Two-component sensor		3		
PA0179		Two-component response regulator				3.258
PA0180	*cttP*	Chemotactic transducer for trichloroethylene		2.526		
PA0289	*gpuR*	Transcriptional activator		2		
PA0294	*aguR*	Transcriptional regulator		2.065		
PA0306a		Transcriptional regulator		2.432		4.198
**PA0355**	***pfpI***	**Protease**			**2.684**	**6.632**
PA0499		Pilus assembly chaperone	2.852	4.296	3	3.259
PA0520	*nirQ*	Regulatory protein		2.711	3.092	3.513
PA0797		Transcriptional regulator		2		
PA0962	*dps*	DNA-binding stress protein, starved cells		2.586		2.387
**PA1001**	***phnA***	**Anthranilate synthase component I**		**0.257**		**0.183**
PA1179	*phoP*	Two-component response regulator				0.307
PA1180	*phoQ*	Two-component sensor kinase				0.331
PA1347		Transcriptional regulator	2.185	2.074		
PA1603		Transcriptional regulator		2.514		2.73
PA1898	*qscR*	Quorum-sensing control repressor		2.25	2.406	2.188
PA1930		Chemotaxis transducer		4.118		
PA2016	*liuR*	Regulator of *liu* genes	2.839	4.361		3.078
PA2028		Transcriptional regulator			2.059	
PA2191	*exoY*	Adenylate cyclase		2.348		
PA2259	*ptxS*	Transcriptional regulator		3		
PA2276		Transcriptional regulator		3.333		
PA2788		Chemotaxis transducer				2.549
**PA3120**	***leuD***	**Isopropylmalate dehydratase**		**2.180**		
PA3477	*rhlR*	Transcriptional regulator		2.411		2.224
PA3757	*nagR*	Transcriptional regulator		3.294		
PA3895		Transcriptional regulator		2.065		
**PA3916**	***moaE***	**Molybdopterin-converting factor**				**0.217**
PA4296	*pprB*	Two-component response regulator		2.538	2.442	4.038
PA4309	*pctA*	Chemotactic transducer		2.273		2.273
PA4499	*psdR*	Transcriptional regulator		2.69		
PA4659		Transcriptional regulator		2.565	3.348	3.739
PA4876	*osmE*	*osmE* family transcriptional regulator				3.742
PA4878	*brlR*	Transcriptional regulator	2.707	2.576	2.22	2.271
PA4915		Chemotaxis transducer		2.5		3.75
PA5356	*glcC*	DNA-binding transcriptional regulator		2.37		
PA5365	*phoU*	Phosphate uptake regulatory protein		2.182	2.864	2.545

a Fold changes of genes at the indicated time points compared to the 5-min sample, where a fold change of 1 indicates no difference, a fold change of >1 indicates genes with elevated RNA levels compared to those at 5 min, and a fold change of <1 indicates genes with reduced RNA levels compared to those at 5 min (underlined). Empty cells indicate that there is no significant difference in gene expression compared to that at 5 min. Boldface type indicates the genes for which follow-up biofilm and complementation assays were performed (Fig. 5).

**FIG 3:**
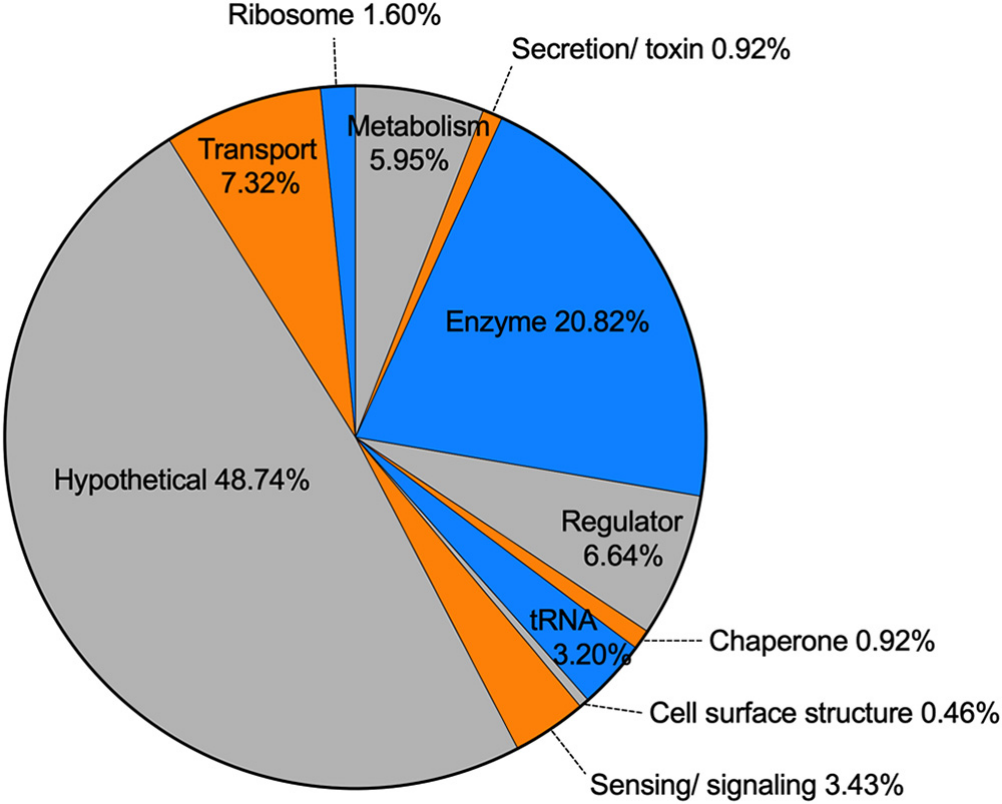
Surface contact results in the regulation of broad classes of genes. The 453 genes with surface-dependent differential regulation were sorted based on gene ontology terms. The percentage of each GO term is indicated.

The organization of the surface-regulated genes by gene ontology (GO) (25, 26) reveals the gene classes most affected by the transition to a surface-associated lifestyle (Fig. 3). The most affected category is hypothetical genes, at 48.7% of the differentially regulated genes. This is not surprising and likely holds many interesting candidates; however, it is beyond the scope of this study to determine the role of these genes. This group does provide promise that novel surface-associated biofilm genes can be identified, potentially encoding new sensors, adhesins, or signaling molecules. Genes encoding enzymes comprise 20.8% of the surface-regulated genes. The next most surface-regulated genes are those involved in transport (7.3%), regulation (6.6%), and metabolism (5.9%). The latter groups indicate the importance of changing metabolic needs upon surface attachment.

What is most striking about these changes is the kinetics. At 30 to 60 min postattachment, the cells appear as individual cells or small clusters and are likely switching their metabolic program to resemble biofilm cells more than planktonic cells. This echoes the findings of Connell et al., who observed that clusters of about 150 cells shared resistance phenotypes with biofilms, indicating that the phenotypic change to biofilms occurs relatively soon after attachment or aggregation (27). Here, we observe that the surface-induced transcriptional changes occur within 1 h of surface attachment. This rapid response indicates that biofilm phenotypes may occur more rapidly than previously proposed and that the phenotype may be triggered by surface contact.

### Surface-regulated genes contribute to biofilm formation.

**FIG 4:**
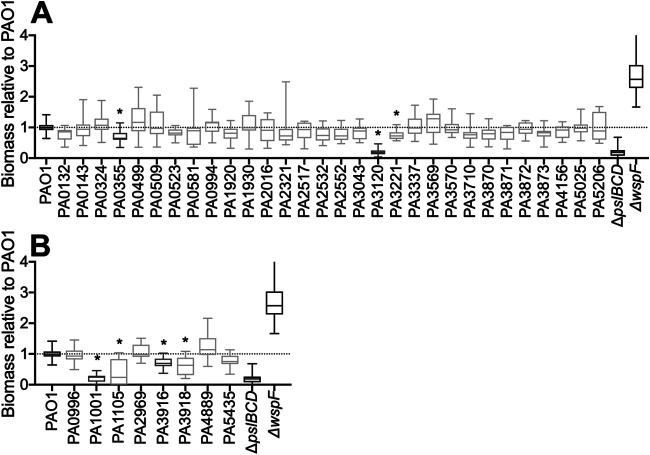
Transposon mutants of genes regulated upon surface attachment caused decreases in biofilm development. Biofilm assays were performed for transposon mutants of 40 of the most significantly differentially regulated genes. This corresponded to 32 genes that had elevated RNA levels (A) and 8 genes that had reduced RNA levels (B). Mutants with transposon insertions in these genes were allowed to adhere to the wells of a 96-well plate, after which the biomass was quantified by crystal violet staining. The biomass was normalized to that of the PAO1 parent strain, which was set to 1. Δ*pslBCD* and Δ*wspF* mutants were used as biofilm-deficient and hyperbiofilm controls, respectively. * indicates a *P* value of <0.05. Data are presented as a box-and-whisker plot of results from 4 biological replicates, each with 4 technical replicates. Genes depicted in dark gray were selected for further analysis (PA0355 [*pfpI*], PA1001 [*phnA*], PA3120 [*leuD*], and PA3916 [*moaE*]).

We posited that genes regulated upon surface attachment are involved in surface sensing and the transition from a motile to a sessile lifestyle. In order to test this hypothesis, mutants of the 40 most significantly differentially surface-regulated genes, identified from the RNA-Seq analysis, were selected from the PAO1 transposon mutant library (28, 29), and the surface attachment of these mutants was assessed relative to the parent PAO1 strain (Fig. 4). This corresponded to 32 genes that had elevated RNA levels (Fig. 4A) and 8 genes that had reduced RNA levels, compared to the 5-min attached population (Fig. 4B). PAO1 Δ*pslBCD* and PAO1 Δ*wspF* mutants were included as controls, as these mutants display defective and hyperbiofilm phenotypes, respectively (14, 30). These assays confirmed that a subset of surface-regulated genes is involved in surface attachment and biofilm initiation, with some mutants displaying biofilm formation comparable to that of the PAO1 Δ*pslBCD* mutant control (Fig. 4). Together, these data validate the observations from the RNA-Seq analysis.

**FIG 5:**
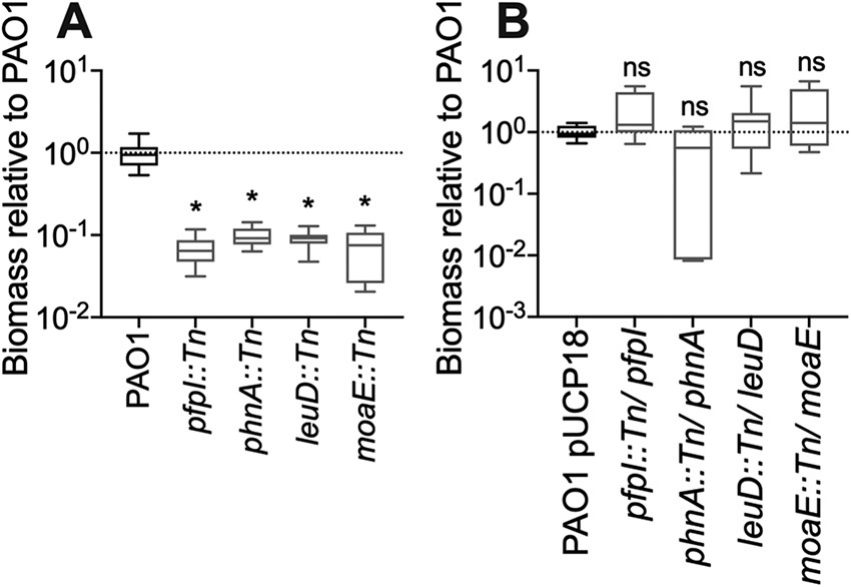
Complementation of the biofilm-deficient phenotype of the identified mutants. Four transposon mutants that displayed significantly reduced biofilms (Fig. 4) (PA0355 [*pfpI*], PA1001 [*phnA*], PA3120 [*leuD*], and PA3916 [*moaE*]) were selected for follow-up biofilm analysis. (A) Biofilms of these mutants were grown on plastic beads for 48 h, and the biomass was quantified by CFU. The biomass was normalized to that of the parent PAO1 strain. (B) The wild-type allele of each transposon-disrupted gene was introduced in *trans* into the respective transposon mutants. Biofilms were grown for 48 h, and the biomass was quantified by CFU. The biomass was normalized to that of the parent PAO1 strain harboring the empty vector pUCP18. * indicates a *P* value of <0.05. ns, not significant. Data are presented as box-and-whisker plots of results from 3 biological replicates, each with 3 technical replicates.

Of the subset of mutants that displayed defective surface attachment, 4 were selected for further analysis: *pfpI* (PA0355), *phnA* (PA1001), *leuD* (PA3120), and *moaE* (PA3916) (Fig. 4, dark gray, and Table 1, boldface type). The biofilm formation capabilities of these mutants were further evaluated using the biofilm bead model (31, 32). This model incorporates all stages of *in vitro* biofilm development: attachment, biofilm growth, dispersal, and attachment to a new surface. As such, this model is robust in assessing the biofilm capacity of bacteria. Using this model, the deficient surface attachment phenotype of the 4 mutants (Fig. 4) manifested as a significantly reduced biofilm biomass compared to that of the parent PAO1 strain (Fig. 5A). To determine if the reduced-biofilm phenotype was due to a disruption of the indicated gene or polar effects of the transposon insertion, the wild-type allele of either *pfpI*, *phnA*, *leuD*, or *moaE* was introduced into the respective transposon mutant in *trans*, and the biofilm bead model was used to assess biofilm biomass levels, compared to the parent PAO1 strain containing the empty vector (pUCP18). For all 4 mutants, the introduction of the wild-type allele restored biofilm biomass levels comparable to those of the parent PAO1 strain (Fig. 5B), confirming that all 4 genes are implicated in biofilm formation. Given that all 4 gene products have roles in metabolism, the growth of the transposon mutants was assessed to determine if growth defects accounted for the reduced-biofilm phenotype. No growth differences were observed for *pfpI* and *moaE* transposon mutants compared to the parent PAO1 strain (Fig. S2A). While *phnA* and *leuD* transposon mutants appeared to have delayed exponential growth in rich and minimal media (Fig. S2A), quantification of the biofilm biomass extending beyond this growth delay revealed that both mutants retained the reduced-biofilm phenotype (Fig. S2B). This suggests that differences in growth do not influence the biofilm phenotype of these mutants.

We posited that genes regulated upon surface contact play a role in surface sensing and the transition from a planktonic to a biofilm lifestyle. In support of this hypothesis, *pfpI*, *phnA*, *leuD*, and *moaE* were all differentially regulated upon surface contact; both *pfpI* and *leuD* had elevated RNA levels, while *phnA* and *moaE* had reduced RNA levels. Consistent with our observations here, these 4 genes have all been implicated in biofilm formation. This indicates that transcriptional responses that are initiated upon surface attachment are critical to and propagated during biofilm development. *pfpI* (PA0355) showed elevated expression at 45 and 60 min postattachment (Table 1). *pfpI* encodes a protease that has been implicated in motility, biofilm formation, antibiotic resistance, and protection against stress responses (33, 34). This suggests that general stress protection mechanisms are activated during the early stages of surface attachment and colonization and are necessary throughout biofilm development. *leuD* (PA3120) showed elevated expression at 30 min postattachment (Table 1). *leuD* encodes an isopropylmalate dehydratase necessary for leucine biosynthesis. Similar to our observations, *leuD* is upregulated in Escherichia coli biofilms and is S-nitrosylated specifically in biofilms grown under anaerobic conditions, along with other proteins required for amino acid synthesis (35). The identification of biofilm-specific S-nitrosylated proteins suggests that reversible redox protein modifications could function as important regulation mechanisms during biofilm growth (35). This is further corroborated by our observation here that a *leuD* mutant displayed reduced biofilm formation (Fig. 4 and 5). Furthermore, *leuD* has been implicated in P. aeruginosa antimicrobial resistance (36), a phenotype that is also linked to biofilm formation (37).

*phnA* (PA1001) showed reduced expression at 30 and 60 min postattachment (Table 1). *phnA* is part of the *pqs* operon (38) and, together with *phnB*, encodes an anthranilate synthase required for phenazine biosynthesis, particularly pyocyanin (39), and for the biosynthesis of the Pseudomonas quinolone signal (PQS), one of the quorum sensing pathways within P. aeruginosa (38). Both phenazine (40, 41) and PQS (42) have well-established roles in P. aeruginosa biofilm development, accounting for the observation here that a *phnA* mutant displayed deficient biofilm formation (Fig. 4 and 5). Consistent with our observations here that *phnA* expression is reduced during surface attachment and colonization, *phnA* expression during P. aeruginosa planktonic logarithmic growth is low, with no PhnAB production being identified and *phnA* expression peaking during stationary phase (39). Together, these observations further corroborate the tight regulation of secondary metabolism and quorum sensing during the early stages of biofilm development. Finally, *moaE* (PA3916) showed reduced expression at 60 min postattachment (Table 1). MoaE, together with MoaD, forms the molybdopterin synthase, which is necessary for molybdopterin cofactor biosynthesis (43). Molybdoenzymes play key roles in metabolism and respiration, particularly under oxygen- and nutrient-limiting conditions, and have been implicated in bacterial virulence, including in P. aeruginosa infection models (44–48). Similar to our observations here, a Burkholderia thailandensis *moeA* mutant, which is required for molybdopterin biosynthesis, showed reduced biofilm levels under both aerobic and anaerobic conditions (49). Of note, a P. aeruginosa PA1006 mutant is deficient in biofilm formation (46). PA1006 is a TusA- or SirA-like protein required for nitrate utilization under anaerobic growth and for the homeostasis of molybdopterin biosynthesis (46). Together, these results suggest that being able to utilize multiple metabolic and respiratory pathways is essential during biofilm development.

Combined, these data indicate that the ability to flexibly transition between metabolic and respiratory states during the transition from a planktonic to a biofilm lifestyle is essential and that these pathways are often interconnected and feed into pathways necessary for subsequent biofilm development, whether it be quorum sensing, redox signaling, or protection against stresses.

### The transcriptional response during attachment is surface specific.

Bacteria with a broad range of niches, such as P. aeruginosa, experience many different surfaces as they transition from one environment to the next. The surfaces to be colonized range from soil particles to the lung epithelium, with a vast disparity in the physical and chemical properties as well as stressors that must be overcome to establish a biofilm. As the bacteria encounter these different microenvironments, they must sense, respond to, and bind to the surfaces in order to form a biofilm community.

**FIG 6:**
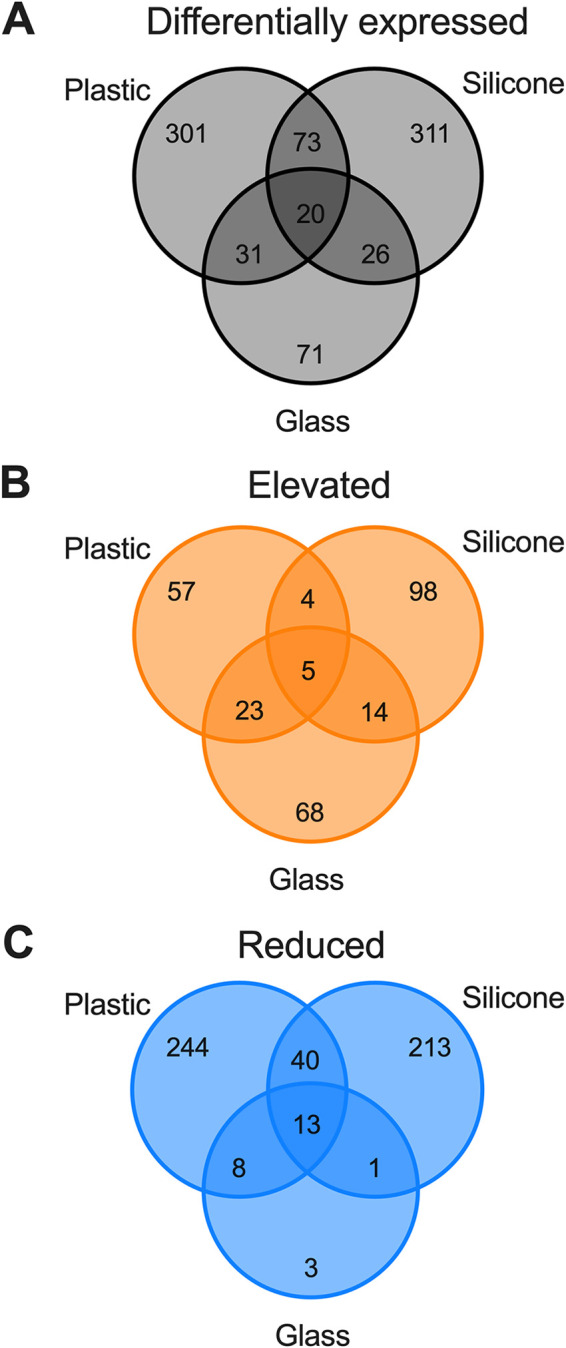
Transcriptional responses are surface specific. Differential expression of genes was determined after 30 min of exposure to the indicated surface (*q* ≤ 0.05). Venn diagrams depict the total number of differentially expressed genes across the indicated surfaces (A) and genes that displayed only elevated (B) or reduced (C) RNA levels across the three surfaces; that is, genes that displayed both elevated and reduced RNA levels across either surface were omitted. The numbers of genes in each group are indicated in the graph.

Our data demonstrated that transcriptional profiling can be used to determine the response to surfaces early after surface attachment. We therefore hypothesized that P. aeruginosa would display a conserved core of transcriptional responses general to abiotic surfaces and responses that were specific to the surface. To test this, we performed RNA-Seq on adherent bacteria recovered from coupons of three medically relevant surfaces, silicone, polycarbonate plastic, and glass, the latter of which is also a widely used surface in *in vitro* biofilm models. Bacteria were allowed to adhere to coupons for 30 min, followed by washing to remove nonadherent bacteria. Thirty minutes was chosen because the transcriptional response from our kinetic analysis peaked at this time point (Fig. 2). RNA was collected, processed, and sequenced from the adherent population as described above (Fig. 1). Sequences were aligned to the PAO1 reference genome, and differential expression was determined using Rockhopper in a pairwise comparison to the 5-min adherent population recovered from the Ibidi μ-Slide. The rationale for this comparison was that this surface was the only sample that had sufficient cell attachment to extract RNA at 5 min postattachment. This comparison is therefore a limitation of the study, as comparisons to the 5-min attached population on the respective surfaces would have been the ideal comparisons. Despite this, these comparisons were sufficient to identify differentially expressed genes. Specifically, we identified a total of 833 genes that were differentially expressed across the three surfaces (Fig. 6A). In contrast to our kinetic analysis (Fig. 2), the expression of most genes was reduced (522 genes) rather than elevated (269 genes), with 42 genes showing both elevated and reduced RNA levels across two or more surfaces (Fig. 6B and C). Contrary to our hypothesis, we observed the regulation of very few genes shared among the different surfaces. Instead, bacteria had a nearly unique transcriptional response on each surface, with the vast majority being regulated on only one surface (Fig. 6; see also Table S2 in the supplemental material).

Only 20 differentially expressed genes were conserved on all three analyzed surfaces (Table 2). Importantly, these included a number of metabolic genes, with some having intersecting roles in both metabolism and biofilm formation, consistent with the genes identified from our kinetic analysis (*pfpI*, *phnA*, *leuD*, and *moaE*). Of particular note, the RNA levels of *moaE* and the remaining genes in the operon, *moaC* and *moaD*, as well as the downstream genes *moeA1* and *moaB1* (PA3914 to PA3918) were reduced across all three surfaces (Table 2). Together, these data indicate that molybdoenzymes may have an important and previously unappreciated role during surface attachment and biofilm formation. In support of this, we also observed nitrogen metabolism genes, specifically *fhp* (PA2664), *narI* (PA3872), and *narK1* (PA3877) (Table 2), that were differentially expressed across all three surfaces. Interestingly, both *narI* and PA3871, a PpiC-type peptidyl-prolyl *cis-trans* isomerase, which are located in the *nar* operon (PA3871 to PA3877), had up to 23-fold-elevated RNA levels (Table 2), indicating the potential importance of regulating nitrogen metabolism in response to surface attachment. Molybdoenzymes play key roles in nitrogen metabolism, particularly in nitric oxide (NO) formation (50, 51). The role of NO signaling in biofilm dispersal is well established in the field (52, 53). However, our results also suggest a role for molybdoenzyme-dependent NO signaling during the early stages of biofilm formation. In support of this, a nitrate-sensing two-component system was found to inhibit Burkholderia pseudomallei biofilm formation (54).

**TABLE 2:** Genes that were differentially expressed across all three surfaces

PA no.	Gene name	Gene product	Fold expression difference (30 min [surface] vs 5 min postattachment^*a*^)		
			Plastic^*b*^	Silicone	Glass
PA0567		Hypothetical protein	0.684	0.200	0.411
PA0623		Bacteriophage protein	0.676	0.214	2.048
PA0839		Transcriptional regulator	0.590	0.000	0.252
PA0996	*pqsA*	Coenzyme A ligase	0.262	0.000	0.274
PA1985	*pqqA*	Coenzyme PQQ synthesis protein PqqA	0.659	0.519	0.317
PA2663	*ppyR*	*psl* and pyoverdine operon regulator, PpyR	0.669	0.000	0.218
PA2664	*fhp*	Nitric oxide dioxygenase	0.164	0.000	0.059
PA2759		Hypothetical protein	4.592	0.398	3.766
PA3205		Hypothetical protein	2.865	2.683	2.746
PA3871		PpiC-type peptidyl-prolyl *cis-trans* isomerase	23.313	2.875	15.000
PA3872	*narI*	Respiratory nitrate reductase subunit gamma	23.238	2.524	13.333
PA3877	*narK1*	Nitrite extrusion protein 1	0.146	0.013	0.123
PA3914	*moeA1*	Molybdenum cofactor biosynthetic protein A1	0.269	0.019	0.298
PA3915	*moaB1*	Molybdopterin biosynthetic protein B1	0.092	0.014	0.070
PA3916	*moaE*	Molybdopterin-converting factor large subunit	0.234	0.127	0.243
PA3917	*moaD*	Molybdopterin-converting factor small subunit	0.282	0.167	0.211
PA3918	*moaC*	Molybdenum cofactor biosynthesis protein MoaC	0.230	0.108	0.225
PA4270.1			2.201	2.007	2.716
PA4637a		Hypothetical protein	0.331	0.209	0.324
PA5369	*pstS*	Phosphate ABC transporter substrate-binding protein	3.353	2.294	3.529

a Fold changes of genes at 30 min postattachment on the indicated surfaces compared to those at 5 min postattachment on an Ibidi μ-Slide, where a fold change of 1 indicates no difference, a fold change of >1 indicates genes with elevated RNA levels compared to those at 5 min, and a fold change of <1indicates genes with reduced RNA levels compared to those at 5 min.

b Polycarbonate plastic.

Consistent with this, we also identified that the RNA levels of *ppyR* were reduced across all three surfaces (Table 2). *ppyR* regulates both the *psl* and *pqs* operons and pyoverdine and anthranilate biosynthesis genes, and a PAO1 *ppyR* mutant has reduced biofilm formation relative to the wild type (55). Interestingly, *ppyR* is part of an operon with PA2662, which is predicted to be an NnrS protein and is downstream of *fhp* (21), also identified in our analysis (Table 2). It has therefore been hypothesized that *ppyR* is a membrane sensor that regulates exopolysaccharide and pyoverdine production through NO signaling (55). Supporting a potential conserved role for *ppyR* during biofilm formation, a screen of 104 P. aeruginosa clinical isolates for the presence of virulence genes identified that 99% of the isolates contained *ppyR* (56). We also identified that *pstS* had elevated RNA levels across all three surfaces (Table 2). PstS is required for phosphate uptake in P. aeruginosa (57). However, mutations in *pstS* that do not affect phosphate binding were found to reduce biofilm formation. It was predicted that PstS plays a structural role in the biofilm, mediating biofilm formation in response to phosphate nutritional cues (58). Together, these data further support our previous conclusion that metabolic pathways may be interconnected and feed into pathways necessary for subsequent biofilm development.

Interestingly, a number of hypothetical proteins were identified, of which PA0567, PA0839, PA2759, and PA3205 (Table 2) have predicted roles in membrane stress and permeability (21). This is suggestive of a conserved role for detecting membrane changes in initiating biofilm formation on abiotic surfaces. Finally, a small noncoding RNA, PA4270.1, was identified that had elevated RNA levels across all three surfaces (Table 2), suggesting that this small RNA may have a conserved role in regulating biofilm formation.

Each of the surfaces tested here is encountered by patients and staff in hospitals and can serve as a reservoir for bacteria, contributing to the persistence of bacteria in the hospital and the transmission of infections. This study has demonstrated that bacteria initiate a rapid, specific transcriptional response to abiotic surfaces that results in attachment and biofilm initiation. From these data, it is clear that bacteria are able to not only sense that they have encountered a surface but also tailor their response to particular properties of each surface. There are differences between these surfaces, including elemental composition, hardness, viscosity, hydrophobicity, and texture. In line with our observations, it has been demonstrated that material stiffness affects bacterial attachment, biofilm formation, and intracellular signaling (59, 60). Proteomic studies of biofilms formed on abiotic surfaces found that there were specific proteomic responses to various surfaces, with the differential detection of 70 of 930 proteins between surfaces (61). This study indicated that members of the proteomes were specifically regulated on different surfaces. It is unclear from these data what exactly the bacteria are sensing; however, future work will attempt to elucidate not only what the bacteria are sensing but also the mechanism of surface sensing and signaling resulting in these drastically different responses to abiotic surfaces as well as identify the roles of the conserved differentially expressed genes during biofilm formation.

### Conclusion.

P. aeruginosa is an example of a significant nosocomial pathogen. Serious P. aeruginosa infections cause an average increase of $16,890 in hospital expenses (62) and increases in hospital lengths of stay by a median of 13 days and 4 times higher for multidrug-resistant P. aeruginosa (63). Therefore, it is essential to identify mechanisms of bacterial persistence and transmission in hospital environments, especially as reports of the prevalence of antimicrobial resistance are increasing.

Biofilms are a mechanism of persistence, contributing to bacterial transmission and infection risk in hospitals, often through association with medical devices and hospital surfaces. Understanding how bacteria respond upon surface attachment to initiate biofilm development is imperative to combating the formation of these persistent microbial communities. Here, we sought to analyze the transcriptional responses of P. aeruginosa across the critical early kinetics of surface adhesion and colonization. We defined the transcriptional responses of P. aeruginosa both across the first hour of surface colonization and across three medically relevant surfaces. These analyses identified that metabolic and respiratory genes that often feed into other pathways necessary for biofilm development are tightly regulated during these early time points, particularly genes important for molybdopterin cofactor biosynthesis and nitrogen metabolism. Importantly, we observed that this transcription response is surface specific, with surfaces commonly encountered in medical settings eliciting unique differential gene expression profiles. This raises the intriguing possibility of combating biofilm colonization of these surfaces by selecting materials in health care settings based on the colonization properties. Another option is engineering surfaces that resist colonization by specific nosocomial pathogens or including materials in the surfaces that prevent recognition by bacteria.

The biofilm model and surfaces analyzed here are specifically relevant to infection of medical devices. A limitation of this study is that these data are not readily transferable to other types of infection such as lung infections in people with CF. Future work will focus on performing similar analyses under other *in vivo*-like growth conditions such as artificial sputum media. However, these analyses set the framework for elucidating the early stages of biofilm formation, which is currently lacking in the field. In light of the proliferation of antimicrobial resistance and the persistence of some infectious diseases, it is imperative to investigate novel approaches to sanitation, infection control, and limitation of biofilm formation. We propose that these studies present an understanding of the initial stages of biofilm formation that may be exploited in the development of these novel infection control strategies.

## MATERIALS AND METHODS

### Bacterial strains and growth conditions.

The bacterial strains used along with genotypes are provided in Table S3 in the supplemental material. Bacterial strains were inoculated into lysogeny broth (LB) (10 g L^−1^ tryptone, 5 g L^−1^ yeast extract, 10 g L^−1^ NaCl) at 37°C for cultures grown overnight in a roller unless otherwise noted. Strains were grown at 37°C on LA (LB solidified with 1.5% agar). Ampicillin at 100 μg/mL and carbenicillin at 300 μg/mL were used to maintain or select for plasmids in E. coli and P. aeruginosa, respectively. Transposon mutants were isolated from the PAO1 Transposon Library (28, 29).

### P. aeruginosa attachment to surfaces.

A logarithmic-phase bacterial culture in LB at an optical density at 600 nm (OD_600_) of 0.5 was inoculated onto an Ibidi μ-Slide, the surface of which is an Ibidi polymer coverslip (catalog number 80606; Ibidi). At the indicated times from 5 to 60 min postattachment, the channel was rinsed twice in phosphate-buffered saline (PBS) to remove nonadherent cells, and 200 μL of TRIzol was added to the channel. TRIzol was collected and stored at −80°C for RNA isolation (Fig. 1). Three independent biological replicates were collected for each time point. For time points with low bacterial adhesion, multiple samples were pooled to generate sufficient RNA for library generation.

Coupons of silicone, glass, and polycarbonate plastic were purchased from Biosurfaces Technologies (catalog numbers RD128-Si, RD128-GL, and RD128-PC). Coupons were placed into a 24-well plate and conditioned in LB for at least 10 min prior to bacterial addition. LB was removed and replaced with 2 mL of a logarithmic-phase bacterial culture in LB (OD_600_ of 0.7). At the indicated time points, the coupon was rinsed twice in PBS and placed into 1 mL of TRIzol. TRIzol was collected and stored at −80°C for RNA isolation. Three independent biological replicates were collected for each surface condition. On surfaces with low bacterial adhesion, multiple samples were pooled to generate sufficient RNA for library generation.

### RNA isolation.

Following a 5-min incubation at room temperature, 0.2 mL of chloroform was added, and the samples were shaken vigorously for 1 min. Phases were separated by centrifugation (12,000 × *g* for 5 min at 4°C), and the aqueous phase was combined with 0.6 mL of 70% ethanol and transferred to an RNeasy minicolumn (Qiagen). RNA was purified according to the manufacturer’s protocols. RNA was eluted in 20 μL of water and stored at −80°C.

### RNA-Seq library construction and sequencing.

RNA quantification was performed on the Qubit 3.0 fluorometer according to the manufacturer’s instructions. Libraries were constructed using the ScriptSeq complete kit (Illumina) according to the bacteria—low-input protocol. Briefly, rRNA was depleted from 100 ng of total RNA with the Ribo-Zero process. rRNA-depleted RNA was fragmented and reverse transcribed using random primers containing a 5′-tagging sequence, followed by 3′-end tagging with a terminus-tagging oligonucleotide to yield di-tagged, single-stranded cDNA. Following purification by a magnetic-bead-based approach, the di-tagged cDNA was amplified by PCR using primer pairs that anneal to tagging sequences, and adaptor sequences required for sequencing cluster generation were added. Amplified RNA-Seq libraries were purified using the AMPure XP system (Beckman Coulter). The quality and quantity of the libraries were determined via an Agilent TapeStation. Sequencing was performed on the Illumina HiSeq 4000 platform.

### RNA-Seq data analysis.

HiSeq 4000 sequencing was performed, generating approximately 300 million total paired-end 300-bp reads from the 15 total samples, with a mean quality score of 37.4. Reads were aligned to the reference PAO1 genome using Rockhopper (22–24). An average of 14.8 million reads aligned to the reference genome (88.8%), with an average of 96.9% aligning to nonribosomal regions. Differential expression analysis was performed by Rockhopper. Gene expression after 30 min was compared to gene expression at 5 min. Differentially expressed genes (*q* ≤ 0.05) were filtered to include only genes with at least 2-fold differential expression.

### Complementation of transposon mutants.

The desired genes were amplified by PCR using primers detailed in Table S4 and genomic DNA isolated from wild-type PAO1. Primers were designed with restriction enzyme sites at the 5′ ends, which are detailed in Table S6. Purified PCR products and empty vector pUCP18 (64) were digested with the appropriate restriction enzymes (New England BioLabs [NEB]) according to the manufacturer’s protocol. Restriction enzymes were heat inactivated by incubation at 80°C for 20 min. The digested PCR products and pUCP18 were ligated using T4 ligase (NEB) according to the manufacturer’s protocol. Five microliters of the ligation reaction mixture was transformed into chemically competent Escherichia coli NEB5α cells and recovered on LA supplemented with 100 μg/mL ampicillin plus 100 μg/mL isopropyl-β-d-thiogalactopyranoside (IPTG) and 40 μg/mL 5-bromo-4-chloro-3-indolyl-β-d-galactopyranoside (X-gal) for blue/white colony selection. Complementing constructs were confirmed by sequencing. Confirmed constructs were purified using a QIAprep spin miniprep kit (catalog number 27106; Qiagen) according to the manufacturer’s protocol. Confirmed constructs were electroporated into the appropriate PAO1 transposon mutants and recovered on LA supplemented with 300 μg/mL carbenicillin.

### Biofilm assays. (i) Microtiter biofilm assay.

Cultures of P. aeruginosa transposon mutants and control strains were grown overnight to mid-logarithmic phase and diluted to an OD_600_ of 0.5 in VBMM (Vogel-Bonner minimal medium) (0.2 g/L MgSO_4_·7H_2_O, 3.5 g/L NaNH_4_HPO_4_·4H_2_O, 10 g/L K_2_HPO_4_, 0.1 g/L CaCl_2_, 2 g/L citric acid, 1 g/L Casamino Acids). One hundred microliters of the normalized culture was transferred to the wells of a 96-well microtiter plate (Corning) and incubated for 3 h at 37°C in a humidified chamber. Wells were washed three times with PBS, and the attached biomass was stained with 120 μL of 0.1% crystal violet for 30 min at room temperature. Biofilms were washed three times with PBS, and bound crystal violet was extracted in 150 μL of ethanol for 30 min at room temperature. The absorbance was then measured on a SpectraMax i3 plate reader (Molecular Devices) at an OD_590_. Absorbance values were normalized to the value for the parent PAO1 strain, which was set to 1. Significance was determined using one-way analysis of variance (ANOVA) with a Dunnett *post hoc* test. Four biological replicates, each with four technical replicates, were performed.

### (ii) Biofilm-coated bead assay.

Cultures of the desired P. aeruginosa strains grown overnight were normalized to an OD_600_ of 1, and 100 μL was transferred to a culture tube containing 5 mL LB and a sterile 7-mm polystyrene bead. Cultures were incubated at 37°C with shaking at 150 rpm for 16 h, after which the biofilm-coated bead was transferred into a new culture tube containing 5 mL LB and a second sterile 7-mm polystyrene bead and incubated at 37°C with shaking at 150 rpm for 16 h. After this second incubation round, the latter biofilm-coated bead was transferred to 1 mL PBS, and the biomass was removed by sonication in a water bath sonicator for 30 s. To fully disaggregate the removed biofilm biomass, the cell suspension was passed through a 22-gauge needle. The resuspended cells were then serially diluted and enumerated for CFU on LA to quantitate the amount of biofilm biomass colonizing the second bead. CFU were then normalized to the value of the parent PAO1 strain, which was set to 1. Three biological replicates were performed, each with three technical replicates. For analysis of the complemented mutants, medium was supplemented with 300 μg/mL carbenicillin. Significance was determined using one-way ANOVA with a Dunnett *post hoc* test. This biofilm assay uses two rounds of biofilm bead growth to encompass all stages of biofilm formation (attachment, biofilm growth, dispersal, and initiation of a new biofilm) (31, 32).

### Data availability.

The data discussed in this publication have been deposited in the NCBI Gene Expression Omnibus (65) and are accessible through GEO series accession numbers GSE194320 (kinetic analysis) and GSE195826 (surface analysis).
