# Supplementary material for: Pseudomonas aeruginosa Initiates a Rapid and Specific Transcriptional Response during Surface Attachment
Source: J Bacteriol. 2022 Apr 25;204(5):e00086-22. doi: 10.1128/jb.00086-22 (PMC9112911; doi:10.1128/jb.00086-22)
Supplement: Supplemental file 1 — supplemental material. Download jb.00086-22-s0001.pdf, PDF file, 1.2 MB [file jb.00086-22-s0001.pdf]

Pseudomonas aeruginosa initiates a rapid and specific transcriptional response during surface attachment

Supplemental Information

Christopher J. Jones, Nikolas Grotewold, Daniel J. Wozniak, Erin S. Gloag

Supplemental Methods

Growth curves

Overnight *P. aeruginosa* cultures were diluted to OD_{600nm} 0.05 in either LB (10 g L^{-1} tryptone, 5 g L^{-1} yeast extract, 10 g L^{-1} NaCl) or VBMM (0.2 g/L $MgSO_4 \cdot 7H_2O$, 3.5 g/L $NaNH_4HPO_4 \cdot 4H_2O$, 10 g/L K_2HPO_4, 0.1 g/L $CaCl_2$, 2g/L citric acid, 1 g/L casamino acid), examples of rich and minimal media respectively. 200 µL was transferred to the wells of a sterile 96-well plate, covered with a sterile breathable membrane. OD_{600nm} was recorded every 30 min for 12 h on a SpectraMax i3 plate reader (Molecular Devices), at 37°C, shaking before each read. 3 biological replicates were performed, each with 4 technical replicates.

Microtitre biofilm assay

Overnight cultures of *P. aeruginosa* strains were grown to mid-logarithmic phase and diluted to an OD_{600nm} 0.5 in VBMM. 100 µL of normalized culture was transferred to the wells of a 96-well microtiter plate (Corning), and incubated for 6 h at 37 ˚C in a humidified chamber. Wells were washed three times with PBS and attached biomass was stained with 120 µL of 0.1% crystal violet for 30 min at room temperature. Biofilms were washed three times with PBS and bound crystal violet was extract in 150 µL of ethanol for 30 min at room temperature. Absorbance was measured on a SpectraMax i3 plate reader (Molecular Devices) at OD_{590nm}. Absorbance values were normalized relative to parent PAO1 strain, which was set to 1. Significance was determined using a one-way ANOVA, with a Dunnett post-hoc test. 3 biological replicates, each with duplicate technical replicates were performed.

Microscopy

GFP-tagged *P. aeruginosa* PAO1 (OD_{600} 0.25) were attached to Ibidi µ Slide IV 0.4 flow chambers for the indicated time (Fig. S1). Nonadherent bacteria were washed away with PBS, fluorescent images acquired on a Nikon A1R confocal microscope, and RNA was isolated with TRIZOL. RNA was purified with the Qiagen RNAeasy kit and quantified with a Qubit 3.0 fluorometer (Fig. S1).

Supplemental Figures

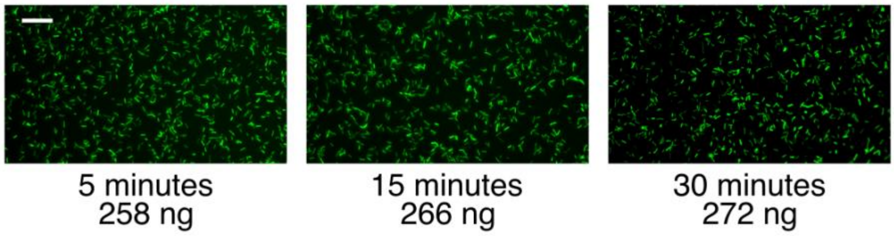

Figure S1: Similar amounts of RNA are recovered at early time points post-attachment. Cells attached to Ibidi μ-Slide were imaged at indicated time points post-attachment, to confirm that the number of attached cells were similar. RNA was extracted from attached cell populations and quantified by Qubit fluorometry. Scale bar indicates 10 μm. Time point post-attachment and RNA concentration is indicated under each representative image.

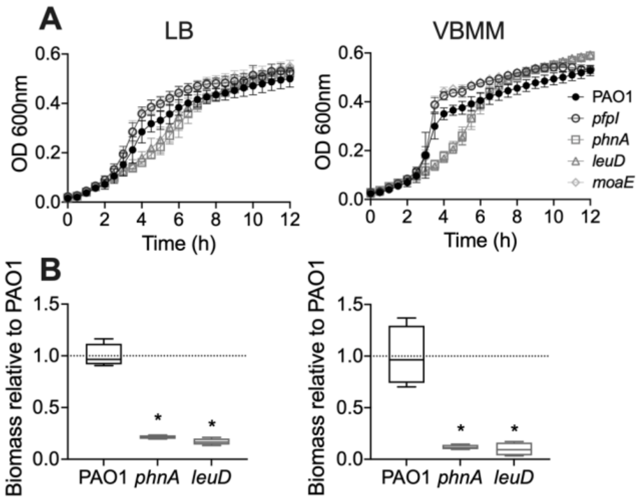

Figure S2: Reduced biofilm phenotype of *phnA* and *leuD* transposon mutants is not due to changes in growth. (A) *P. aeruginosa* transposon mutants were grown in either LB (left panel; labelled) or VBMM (right panel; labelled) for 12 h. OD_{600nm} was recorded every 30 min. In both rich (LB) and minimal media (VBMM) *phnA* and *leuD* transposon mutants appeared to have a delayed exponential phase. Data depicted as mean ± SD of 3 biological replicates, each with 4 technical replicates **(B)** Biofilm assays were performed for *phnA* and *leuD* transposon mutants. Biofilms were grown in either LB (left panel; labelled) or VBMM (right panel; labelled) for 6 h, which was where growth of the mutants reached similar levels to the parent PAO1 strain in A. Biomass was normalized relative to the PAO1 parent strain, which was set to 1. * indicates *p*-value < 0.05. Data is depicted as a box and whisker plot of 3 biological replicates, each with 2 technical replicates.

Supplemental Tables

Table S1: Kinetic surface response to plastic.

PA number	Gene name	Gene product	Gene product class	Fold change compared to 5 min[a]				
				**10 min**	**15 min**	**30 min**	**45 min**	**60 min**
PA0012		hypothetical protein	Hypothetical			2		
PA0034		two-component response regulator	Sensing/ signaling			2.08		
PA0036	*trpB*	tryptophan synthesase subunit beta	Enzyme					2.514
PA0041a					3.713	3.38	2.37	3.38
PA0059	*osmC*	osmotically inducible protein	Cell surface/ Structure				2.667	3.232
PA0103a		hypothetical protein	Hypothetical			2.088		
PA0109		hypothetical protein	Hypothetical			2.46	2.476	3.19
PA0113		protoheme IX farnesyltransferase	Enzyme			2.875		
PA0117		short-chain dehydrogenase	Metabolism			2.067		
PA0126		hypothetical protein	Hypothetical			2.135	2.297	2.216
PA0129	*gabP*	gamma0aminobutyrate permease	Enzyme			2.867		
PA0130		aldehyde dehydrogenase	Enzyme			3.711	4.515	3.68
PA0131		hypothetical protein	Hypothetical		2.237	3.748	3.871	3.144
PA0132		beta alanine-pyruvate transaminase	Enzyme		2.441	4.206	4.324	2.794
PA0153	*pcaH*	protocatechuate 3,4-dioxygenase subunit	Enzyme			2.8		
PA0155	*pcaR*	transcriptional regulator	Regulator			2.56		
PA0159		transcriptional regulator	Regulator		2.474	2	2.368	2.211
PA0160		hypothetical protein	Hypothetical			2.261	2.152	
PA0167		transcriptional regulator	Regulator			2.403		2.235
PA0176	*aer2*	aerotaxis transducer	Sensing/ signaling					2.833
PA0177		purine-binding chemotaxis protein	Sensing/ signaling			3.042		5.75
PA0178		two component sensor	Sensing/ signaling			3		
PA0179		two component response regulator	Sensing/ singaling					3.258
PA0180	*cttP*	chemotactic transducer for trichloroethylene	Sensing/ signaling			2.526		
PA0200		hypothetical protein	Hypothetical		3.226	3.058	3.371	3.972
PA0226		CoA tranferase, subunit A	Enzyme			2.8		
PA0234		hypothetical protein	Hypothetical		2.2	2.267	2.933	
PA0250		hypothetical protein	Hypothetical					2.342
PA0256		hypothetical protein	Hypothetical					2.31
PA0263	*hcpC*	secreted protein hcp	Secretion/ toxin			2.398		
PA0283	*sbp*	sulfate-binding protein	Transport			2.444		
PA0289	*gpuR*	transcriptional activator	Regulator			2		
PA0293	*aguB*	N-carbamoylputrescine amidohydrolase	Enzyme			2.703		
PA0294	*aguR*	transcriptional regulator	Regulator			2.065		
PA0295		periplasmic polyamine binding protein	Transport			2.143		

PA0297	*spuA*	glutamine amidotransferase	Enzyme		2.737	3.538		
PA0306a		transcriptional regulator	Regulator			2.432		4.198
PA0323		ABC transporter	Transport			2.5		
PA0324		ABC transporter permease	Transport			3.905	3.905	3.429
PA0346		hypothetical protein	Hypothetical					0.329
PA0355	*pfpI*	protease	Enzyme				2.684	6.632
PA0365		hypothetical protein	Hypothetical			2.45		
PA0398		hypothetical protein	Hypothetical					2.098
PA0405		hypothetical protein	Hypothetical			2.55		
PA0442		hypothetical protein	Hypothetical			2.806		2.645
PA0470	*fiuA*	ferrichrome receptor	Sensing/ signaling			2.421		
PA0484		hypothetical protein	Hypothetical			2.087		3.043
PA0492		hypothetical protein	Hypothetical			2.261	3.148	3.458
PA0493		hypothetical protein	Hypothetical		2.489	2.88	2.891	3.435
PA0494		acetyl-CoA carboxylase biotin carboxylase subunit	Enzyme			2.138	3.276	3.31
PA0495		hypothetical protein	Hypothetical			2.885	3.885	3.538
PA0496		hypothetical protein	Hypothetical				3.6	3.1
PA0497		hypothetical protein	Hypothetical		2.794		2.618	2.529
PA0498		hypothetical protein	Hypothetical		2.184	2.388	2.245	2.51
PA0499		pili assembly chaperone	Chaperone		2.852	4.296	3	3.259
PA0505		hypothetical protein	Hypothetical					2.346
PA0509	*nirN*	cytochrome C	Metabolism		3.417	4.979	3.604	4.083
PA0510		uroporphyrin-III C-methyltransferasse	Enzyme			2.871	2.419	3.161
PA0520	*nirQ*	regulatory protein	Regulator			2.711	3.092	3.513
PA0521		cytochrome C oxidase subunit	Enzyme				2.948	2.914
PA0522		hypothetical protein	Hypothetical					2.085
PA0523	*norC*	nitric-oxide reductase subunit C	Enzyme			5.89		
PA0525	*norD*	dinitrification protein	Metabolism				2.337	2.946
PA0532		hypothetical protein	Hypothetical				4.2	
PA0567		hypothetical protein	Hypothetical					3.448
PA0573		hypothetical protein	Hypothetical		2.343	3.086	2.143	2.143
PA0574.1		Met tRNA	Metabolism					0.272
PA0578		hypothetical protein	Hypothetical					0.35
PA0580	*gcp*	DNA-binding/iron metalloprotein/AP endonucleaseEnzyme						0.366
PA0581	*plsY*	glycerol-3-phosphate acyltransferase	Enzyme					2.325
PA0582	*folB*	dihydroneopterin aldolase	Enzyme			0.356		
PA0586		hypothetical protein	Hypothetical					2.696
PA0587		hypothetical protein	Hypothetical			2.271		3.322
PA0588		hypothetical protein	Hypothetical			2.414		3.539
PA0603		ABC transporter ATP-binding protein	Transport					3.042
PA0614		hypothetical protein	Hypothetical				2.872	
PA0617		bacteriophage protein	Phage				2.727	
PA0618		bacteriophage protein	Phage				2.553	

PA0619		bacteriophage protein	Phage				2.5	
PA0621		hypothetical protein	Hypothetical				2.349	
PA0623		bacteriophage protein	Phage				2.279	2.098
PA0624		hypothetical protein	Hypothetical				2.186	
PA0628		hypothetical protein	Hypothetical				2.412	
PA0633		hypothetical protein	Hypothetical				2.339	
PA0634		hypothetical protein	Hypothetical				2.241	
PA0635		hypothetical protein	Hypothetical				2.31	2.241
PA0636		hypothetical protein	Hypothetical				2.475	
PA0639		hypothetical protein	Hypothetical				3.077	2.769
PA0640		bacteriophage protein	Phage				2.8	
PA0641		bacteriophage protein	Phage				2.656	
PA0646		hypothetical protein	Hypothetical				2.245	
PA0656		HIT family protein	Enzyme			2.633		3.833
PA0668.5		5S ribosomal RNA	rRNA			2.735		
PA0726		hypothetical protein	Hypothetical		2.667	2.667		
PA0729.1		Gly tRNA	tRNA			2.035		
PA0732		hypothetical protein	Hypothetical			2.514		
PA0734		hypothetical protein	Hypothetical			1.921		
PA0745		enoyl-CoA hydratase	Enzyme			2.817	2.133	2.633
PA0746		acyl-CoA dehydrogenase	Enzyme			3.167		
PA0747		aldehyde deydrotenase	Enzyme		2.4	2.24		
PA0788		hypothetical protein	Hypothetical		3.462			
PA0797		transcriptional regulator	Regulator			2		
PA0807	*ampDh3*	protein AmpDh3	Enzyme				2.058	
PA0821		hypothetical protein	Hypothetical		2.326	2.512	2.116	2.14
PA0827		hypothetical protein	Hypothetical			2.286		
PA0862		hypothetical protein	Hypothetical			2.061	2.306	2.796
PA0865	*hpd*	4-hydroxyphenylpyruvate dioxygenase	Enzyme			2.851		
PA0869	*pbpG*	D-alanyl-D-alanine endopeptidase	Enzyme			2.302		2.111
PA0871	*phhB*	pterin-4-alpha-carbinolamine deydratase	Enzyme			2.06		
PA0880		ring cleaving dioxygenase	Enzyme			3.214		
PA0887	*acsA*	acetyl-CoA synthetase	Enzyme			2.771	2.543	3.8
PA0911		hypothetical protein	Hypothetical			2.833		
PA0918		cytochrome b561	Metabolism			2.254	2.262	3.09
PA0921		hypothetical protein	Hypothetical					0.392
PA0962		DNA-binding stress protein	Chaperone			2.586		2.387
PA0996	*pqsA*	coenzyme A ligase	Enzyme					0.183
PA1001	*phnA*	anthranilate synthase component I	Enzyme			0.257		0.183
PA1034		hypothetical protein	Hypothetical			2.494		
PA1040		hypothetical protein	Hypothetical			2.206		
PA1041		hypothetical protein	Hypothetical			2.774		4.915
PA1049	*pdxH*	pyridoxamine 5'-phosphate oxidase	Enzyme			2.688		2.969
PA1065		hypothetical protein	Hypothetical			1.96		
PA1076		hypothetical protein	Hypothetical					2.398
PA1172	*napC*	cytochrome C protein	Metabolism			2.069		3.172

PA1177	*napE*	periplasmic nitrate reductase protein	Enzyme				2.893
PA1179	*phoP*	two component response regulator	Sensing/ signaling				0.307
PA1180	*phoQ*	two component sensor kinase	Sensing/ signaling				0.331
PA1197		NAD-dependent deacetylase	Enzyme		2.359		
PA1218		hypothetical protein	Hypothetical		3.813		
PA1289		hypothetical protein	Hypothetical		3.571		5
PA1302		heme utilization protein	Metabolism		2.474		
PA1322		TonB dependent receptor	Sensing/ signaling		3.5		
PA1323		hypothetical protein	Hypothetical				2.426
PA1324		hypothetical protein	Hypothetical			2.568	5.432
PA1324.1				2.346	3.636		
PA1333		hypothetical protein	Hypothetical		2.478	2.174	3.196
PA1343		hypothetical protein	Hypothetical				0.335
PA1347		transcriptional regulator	Regulator	2.185	2.074		
PA1358		hypothetical protein	Hypothetical		2.061		
PA1362		hypothetical protein	Hypothetical		2.5		
PA1368		hypothetical protein	Hypothetical	2.185			
PA1384	*galE*	UDP-glucose 4-epimerase	Enzyme	2.69			
PA1385		glycosyl transferase family protein	Enzyme	2.289			
PA1386		ABC transporter ATP-binding protein	Transport	2.531			
PA1388		hypothetical protein	Hypothetical		2.476		
PA1393	*cysC*	adenosine 5'-phosphosulfate (APS) kinase	Enzyme	2.259	2.037	2.741	
PA1420		hypothetical protein	Hypothetical		3.944	2.338	3.085
PA1421	*gbuA*	guanidinobutyrase	Enzyme		2.542		
PA1477	*ccmC*	heme exporter protein	Transport				0.338
PA1501		hypothetical protein	Hypothetical		7		
PA1561	*aer*	aerotaxis receptor	Sensing/ signaling		3.178		2.712
PA1603		transcriptional regulator	Regulator		2.514		2.73
PA1604		hypothetical protein	Hypothetical	2.37	2.037	2.741	3.389
PA1618		hypothetical protein	Hypothetical				2.109
PA1632	*kdpF*	potassium-transporting ATPase F	Transport		2.579		
PA1647		sulfate transporter	Transport		3.235		
PA1673		hypothetical protein	Hypothetical				3.564
PA1693	*pscR*	type III secretion system protein	Secretion/ toxin	2.371	2.329		2.1
PA1774	*crfX*	crfX protein	Hypothetical				0.24
PA1784		hypothetical protein	Hypothetical		4.941		
PA1796.2		His tRNA	tRNA				0.321
PA1796.3		Leu tRNA	tRNA				0.252
PA1796.4		His tRNA	tRNA				0.293
PA1829		hypothetical protein	Hypothetical		2.259		
PA1830		hypothetical protein	Hypothetical				0.349
PA1837a		hypothetical protein	Hypothetical	3.694	3.194	2.323	2.484
PA1852		hypothetical protein	Hypothetical				0.24

PA1863	*modA*	molybdate-binding periplasmic protein	Transport		2		
PA1869		acyl carrier protein	Transport		0.354		
PA1881		oxidoreductase	Enzyme		2.636		
PA1895		hypothetical protein	Hypothetical		3.389		
PA1898	*qscR*	quorum-sensing control repressor	Regulator		2.25	2.406	2.188
PA1920	*nrdD*	anaerobic ribonucleoside triphosphate reductase	Enzyme		5		
PA1930		chemotaxis transducer	Sensing/ signaling		4.118		
PA1940		hypothetical protein	Hypothetical			2.607	
PA1942		hypothetical protein	Hypothetical		2.559		
PA1943		hypothetical protein	Hypothetical		3.5		
PA1954		hypothetical protein	Hypothetical		3.737		
PA1963		hypothetical protein	Hypothetical		2.364		
PA1970		hypothetical protein	Hypothetical		2.625		2.306
PA1984	*exaC*	NAD+ dependent aldehyde dehydrogenase	Enzyme		2.036		
PA1996	*ppiC1*	peptidyl-prolyl cis-trans isomerase C1	Enzyme	2.208			
PA2003	*bdhA*	3-hydroxybutyrate dehydrogenase	Enzyme		2.407		
PA2009	*hmgA*	homogentisate 1,2-dioxygenase	Enzyme		2.207		
PA2016	*liuR*	regulator of liu genes	Regulator	2.839	4.361		3.078
PA2028		transcriptional regulator	Regulator			2.059	
PA2036		hypothetical protein	Hypothetical	2.349			
PA2040		glutamine synthesis	Metabolism		2.365		
PA2041		amino acid permease	Transport		2.478		
PA2045		hypothetical protein	Hypothetical		2.104		
PA2101		hypothetical protein	Hypothetical	2.6			
PA2102		hypothetical protein	Hypothetical	2.441		2.294	2.265
PA2116		hypothetical protein	Hypothetical		2.889		3.259
PA2118a		hypothetical protein	Hypothetical		2.004		2.658
PA2119		alcohol dehydrogenase	Enzyme		2.74	2.532	3.273
PA2146		hypothetical protein	Hypothetical			2.438	
PA2166		hypothetical protein	Hypothetical				11.538
PA2174		hypothetical protein	Hypothetical		2.609		
PA2190		hypothetical protein	Hypothetical		2.243	2.541	2.054
PA2191	*exoY*	adenylate cyclase	Secretion/ toxin		2.348		
PA2222		hypothetical protein	Hypothetical	2.403			
PA2259	*ptxS*	transcriptional regulator	Regulator		3		
PA2276		transcriptional regulator	Regulator		3.333		
PA2321		gluconokinase	Enzyme			2.068	
PA2367		hypothetical protein	Hypothetical		2.857		
PA2381		hypothetical protein	Hypothetical		3.467	2.478	3.543
PA2397	*pvdE*	pyoverdine biosynthesis protein	Metabolism	2.147	3.792	3.75	
PA2423		hypothetical protein	Hypothetical		2.3		2.725
PA2433		hypothetical protein	Hypothetical				4.179
PA2485		hypothetical protein	Hypothetical		2.153		2.176
PA2486		hypothetical protein	Hypothetical		2.797	2.77	3.935
PA2504		hypothetical protein	Hypothetical		2.25		5.3

PA2513	*antB*	anthranilate dioxygenase small subunit	Enzyme			4.071		
PA2517	*xylY*	toluate 1,2-dioxygenase subunit beta	Enzyme			6.2		
PA2553		acyl-CoA thiolase	Enzyme			2.568	2.892	2.973
PA2554		short-chain dehydrogenase	Enzyme			2.918	3.164	3.41
PA2555		AMP-binding protein	Metabolism		2.417	2.576	2.444	2.364
PA2619	*infA*	translation initiation factor IF-1	Metabolism					0.253
PA2663	*ppyR*	psl and pyoverdine operon regulator	Regulator			2.227		2.386
PA2679		hypothetical protein	Hypothetical					5.667
PA2709	*cysK*	cysteine synthase A	Enzyme			2.23		
PA2736.1		Pro tRNA	tRNA		2.384	2.308		
PA2747		hypothetical protein	Hypothetical			3.039	5.68	7
PA2753		hypothetical protein	Hypothetical		2.372			2.349
PA2754		hypothetical protein	Hypothetical		2.221	2.767	2.313	3.824
PA2759		hypothetical protein	Hypothetical				2.398	3.805
PA2776		hypothetical protein	Hypothetical			2.069		
PA2781		hypothetical protein	Hypothetical					2.216
PA2788		chemotaxis transducer	Sensing/ signaling					2.549
PA2790		hypothetical protein	Hypothetical			2.538		
PA2805		hypothetical protein	Hypothetical			2.724		2.952
PA2819.2		Gly tRNA	tRNA					0.312
PA2819.3		Glu tRNA	tRNA					0.317
PA2840		ATP-dependent RNA helicase	Enzyme					0.328
PA2864		hypothetical protein	Hypothetical					2.135
PA2918		short-chain dehydrogenase	Enzyme					2.355
PA2937		hypothetical protein	Hypothetical					2.411
PA2942.1						3.037		2.354
PA2968	*fabD*	malonyl CoA-ACP transacylase	Enzyme					0.294
PA2969	*plsX*	glycerol-3-phosphate acyltransferase	Enzyme					0.251
PA3017		hypothetical protein	Hypothetical					2.704
PA3035		glutathione S-transferase	Enzyme			2.211		
PA3038		porin	Transport		2.337	2.436		2.745
PA3049	*rmf*	ribosome modulation factor	Metabolism		2.305	4.874	5.388	10.209
PA3054		hypothetical protein	Hypothetical			3.375		
PA3109		hypothetical protein	Hypothetical					0.323
PA3120	*leuD*	isopropylmalate dehydratase				2.180		
PA3133.1		Glu tRNA	tRNA					0.196
PA3133.2		Ala tRNA	tRNA					0.22
PA3133.3		Glu tRNA	tRNA					0.184
PA3133.4		Ala tRNA	tRNA					0.169
PA3229		hypothetical protein	Hypothetical			3.535	2.606	4.167
PA3234		acetate permease	Enzyme			3.692		5.846
PA3235		hypothetical protein	Hypothetical			4.636	5.545	7.709
PA3238		hypothetical protein	Hypothetical			2.364	2.273	
PA3255		hypothetical protein	Hypothetical			2.174		

PA3337	*rfaD*	ADP-L-glycero-D-manno-heptose-6-epimerase	Enzyme		3.944	3.495	5.026
PA3342		hypothetical protein	Hypothetical		2.034		2.759
PA3363	*amiR*	aliphatic amidase regulator	Regulator				2.08
PA3365		chaperone	Chaperone		2.156	2.356	2.667
PA3366	*amiE*	acylamide amidohydrolase	Enzyme				2.903
PA3366.1	*amiL*	ncRNA	Hypothetical	2.436	2.621		
PA3369		hypothetical protein	Hypothetical	3.041	2.979	8.383	8.872
PA3370		hypothetical protein	Hypothetical			7.071	8.571
PA3395	*nosY*	ABC transporter	Transport		2.095		
PA3396	*nosL*	NosL protein	Metabolism		3.132	2.5	3.053
PA3418	*ldh*	leucine dehydrogenase	Enzyme		3.071		5
PA3427		short-chain dehydrogenase	Enzyme		2.389		
PA3451		hypothetical protein	Hypothetical		3.857	3.429	7.048
PA3465		hypothetical protein	Hypothetical		3.054	2.216	3.081
PA3477	*rhlR*	transcriptional regulator	Regulator		2.411		2.224
PA3519		hypothetical protein	Hypothetical		3.286		
PA3520		hypothetical protein	Hypothetical	2.61			
PA3530		hypothetical protein	Hypothetical			3.042	
PA3553	*arnC*	Amino sugar and nucleotide sugar metabolism	Metabolism				0.366
PA3566		hypothetical protein	Hypothetical		1.967		
PA3557	*arnE*	Lipid metabolism	Metabolism				0.33
PA3566		hypothetical protein	Hypothetical				2.067
PA3569	*mmsB*	3-hydroxyisobutyrate dehydrogenase	Enzyme		7		
PA3570	*mmsA*	methylmalonate-semialdehyde dehydrogenase	Enzyme	2.811	4.514	4.543	4.4
PA3572		hypothetical protein	Hypothetical		2.333	3.319	5.519
PA3577		hypothetical protein	Hypothetical	3.457	3.159		2.444
PA3614		hypothetical protein	Hypothetical	2.681	3.705	3.013	3.692
PA3615		hypothetical protein	Hypothetical		3.052	2.442	3.403
PA3660		sodium/ hydrogen aniporter	Transport		2.083		
PA3661		hypothetical protein	Hypothetical		4.591		
PA3662		hypothetical protein	Hypothetical		2.006		
PA3691		hypothetical protein	Hypothetical				3.236
PA3692	*lptF*	lipotoxin F	Secretion/ toxin				3.022
PA3710		GMC-type oxidoreductase	Enzyme		5		
PA3712		hypothetical protein	Hypothetical			2.561	3.509
PA3738	*xerD*	site-specific tyrosine recombinase	Enzyme		2.448		
PA3745	*rpsP*	30s Ribosomal protein S16	Ribosome				0.249
PA3757		transcriptional regulator	Regulator		3.294		
PA3786		hypothetical protein	Hypothetical		2.278		
PA3795		oxidoreductase	Enzyme			2.116	2.581
PA3824.1		Leu tRNA	tRNA				0.369
PA3835		hypothetical protein	Hypothetical	2.159			
PA3842		chaperone	Chaperone	3.115		2.075	2.15

PA3857	*pcs*	phosphatidylcholine synthase	Enzyme			2.375		2.2
PA3858		amino acid binding protein	Transport					3.485
PA3859		carboxylesterase	Enzyme			2.357		
PA3865a		hypothetical protein	Hypothetical			2.062		
PA3867		DNA invertase	Enzyme			2.643	2.321	2.339
PA3869		hypothetical protein	Hypothetical		2.235	2.37	2.5	2.435
PA3870	*moaA1*	molybdenum cofactor biosynthesis protein A	Metabolism	5	2.025	7.28	6.6	3.04
PA3871		PpiC-type peptidyl-prolyl cit-trans isomerase	Enzyme	5		7.733	7.333	3.483
PA3872	*narI*	respiratory nitrate reductase subunit gamma	Enzyme	5	2.217	12.115	7.77	4.852
PA3873	*narJ*	respiratory nitrate reductase subunit delta	Enzyme	5	4.28	8	7.36	4.76
PA3874	*narH*	respiratory nitrate reductase subunit beta	Enzyme		4.7	3.395	2.796	
PA3876	*narK2*	nitrate extrusion protein 2	Transport					0.135
PA3877	*narK1*	nitrate extrusion protein 1	Transport				0.189	0.097
PA3895		transcriptional regulator	Regulator			2.065		
PA3911		hypothetical protein	Hypothetical					0.213
PA3912		hypothetical protein	Hypothetical					0.26
PA3914	*moeA1*	molybdenum cofactor biosynthetic protein A1	Metabolism				0.145	0.073
PA3915	*moaB1*	molybdopterin biosynthetic protein B1	Metabolism				0.153	0.096
PA3916	*moaE*	molbdopterin converting factor large subunit	Metabolism					0.217
PA3917	*moaD*	molybdopterin converting factor small subunit	Metabolism					0.185
PA3918	*moaC*	molybenum cofactor biosynthesis protein	Metabolism				0.218	0.206
PA3919		hypothetical protein	Hypothetical		5.033	6.762	6.024	9.429
PA3922		hypothetical protein	Hypothetical			3.45		
PA3923		hypothetical protein	Hypothetical			3.45		4.05
PA3924		long chain fatty acid CoA ligase	Enzyme			3.692		
PA3945		hypothetical protein	Hypothetical					2.098
PA3986		hypothetical protein	Hypothetical					3
PA4015		hypothetical protein	Hypothetical			2.19		2.431
PA4017		hypothetical protein	Hypothetical					2.25
PA4026		acetyltransferase	Enzyme			2.244	2.171	2.22
PA4033		hypothetical protein	Hypothetical				2.97	2.667
PA4103		hypothetical protein	Hypothetical			3.615		
PA4108a		hypothetical protein	Hypothetical		4.28	2.605	2.209	2.14
PA4115		hypothetical protein	Hypothetical				2.505	
PA4156		TonB dependent receptor	Sensing/ signaling			3.929		
PA4180		acetolactate synthase	Enzyme					2.128
PA4211	*phzB1*	phenazine biosynthesis protein	Metabolism					3.385

PA4242	*rpmJ*	50S ribosomal protein L36	Ribosome					0.315
PA4280.1		5S ribosomal RNA	Ribosome		2.976	2.935		
PA4295	*fppA*	Flp prepilin peptidase A	Enzyme			2.107		
PA4296	*pprB*	two-component response regulator	Regulator			2.538	2.442	4.038
PA4309	*pctA*	chemotactic transducer	Sensing/ signaling			2.273		2.273
PA4327		hypothetical protein	Hypothetical		2.154	3.759	2.69	2.966
PA4328		hypothetical protein	Hypothetical			3.074	2.951	3.099
PA4357		hypothetical protein	Hypothetical			0.322		0.342
PA4432	*rpsI*	30S ribosomal protein S9	Ribosome					0.341
PA4455		ABC transporter permease	Transport					0.382
PA4456		ABC transporter ATP binding protein	Transport					0.286
PA4480	*mreC*	rod shape determining protein	Cell surface structure					0.369
PA4499		transcriptional regulator	Regulator			2.69		
PA4500		ABC transporter	Transport		2.286	2.254		
PA4502		ABC transporter	Transport			3.125		
PA4503		Abc transporter permease	Transport		2.621	3.548		2.935
PA4522	*ampD*	N-acetyl-anhdromuranmyl-L-alanine amidase	Enzyme			1.977		
PA4531		hypothetical protein	Hypothetical					2.284
PA4535		hypothetical protein	Hypothetical					2.127
PA4568	*rplU*	50S ribosomal protein L21	Ribosome					0.173
PA4571		cytochrome C	Metabolism		2.42	3.225	2.416	2.528
PA4575		hypothetical protein	Hypothetical			2.256	2.564	2.59
PA4577		hypothetical protein	Hypothetical					2.573
PA4587	*ccpR*	cytochrome C551 peroxidase	Enzyme			2.631		3.461
PA4590	*pra*	protein activator	Regulator			2.074		
PA4607		hypothetical protein	Hypothetical			2.84		4.405
PA4608		hypothetical protein	Hypothetical					2.553
PA4610		hypothetical protein	Hypothetical		2.452			2.553
PA4611		hypothetical protein	Hypothetical		3.303	5.082	2.897	3.443
PA4616		c4-dicarboxylate-binding protein	Transport					0.298
PA4623		hypothetical protein	Hypothetical			2.861	3.295	4.104
PA4648		hypothetical protein	Hypothetical			3.565		3.783
PA4657		hypothetical protein	Hypothetical					2.333
PA4658		hypothetical protein	Hypothetical		2.026	3.16	4.06	4.58
PA4659		transcriptional regulator	Regulator			2.565	3.348	3.739
PA4660	*phr*	deoxyribodipyrimidine photolyase	Enzyme			2.526	3.947	4.579
PA4669.1		Gln tRNA	tRNA					0.296
PA4673.1		Met tRNA	tRNA		2.441	2.504		
PA4683		hypothetical protein	Hypothetical					0.349
PA4690.1		5S ribosomal RNA	Ribosome		3.292	2.806		
PA4703		hypothetical protein	Hypothetical		2.4	3.263	2.421	5
PA4714		hypothetical protein	Hypothetical					2.241
PA4715		aminotransferase	Enzyme					2.754
PA4724.1		hypothetical protein	Hypothetical			2.464		

PA4738		hypothetical protein	Hypothetical				6.289	15.086
PA4739		hypothetical protein	Hypothetical				6.956	10.776
PA4770	*lldP*	L-lactate permease	Enzyme					2.831
PA4773		hypothetical protein	Hypothetical					0.296
PA4782		hypothetical protein	Hypothetical					0.316
PA4793		hypothetical protein	Hypothetical			3.166		2.597
PA4794		hypothetical protein	Hypothetical			2.829		2.486
PA4826		hypothetical protein	Hypothetical			2.165		
PA4870		hypothetical protein	Hypothetical		2.816	2.2	2.975	3.475
PA4874		hypothetical protein	Hypothetical			2.618		2.977
PA4876	*osmE*	osmE family transcriptional regulator	Regulator					3.742
PA4877		hypothetical protein	Hypothetical					3.4
PA4878		transcriptional regulator	Regulator		2.707	2.576	2.22	2.271
PA4880		bacterioferritin	Metabolism					3.686
PA4887		major facilitator superfamily (MFS) transporter	Transport					0.295
PA4888	*desB*	acyl0CoA desaturase	Enzyme					0.245
PA4889		oxidoreductase	Enzyme					0.271
PA4897		hypothetical protein	Hypothetical			3		
PA4915		chemotaxis transducer	Sensing/ signaling			2.5		3.75
PA4921		hypothetical protein	Hypothetical			0.245		0.255
PA5016	*aceF*	dihydrolipoamide acetyltransferase	Enzyme					0.32
PA5025	*metY*	O-acetylhomoserine aminocarboxypropyltransferase	Enzyme		2.2	4.21	2.516	3.548
PA5026		hypothetical protein	Hypothetical		2.338	2		3.12
PA5027		hypothetical protein	Hypothetical			4.011	3.165	3.813
PA5058		hypothetical protein	Hypothetical			2.381		
PA5073		hypothetical protein	Hypothetical			2.394		
PA5095		ABC transporter permease	Transport			2.1		
PA5103		hypothetical protein	Hypothetical					2.135
PA5105	*hutC*	histidine utilization repressor	Regulator				2.215	2.418
PA5106		N-formimino-L-glutamatedeiminase	Enzyme			5.889		
PA5118	*thiI*	thiamine biosynthesis protein	Metabolism					0.358
PA5139		hypothetical protein	Hypothetical					0.275
PA5151		hypothetical protein	Hypothetical			2.207		
PA5160		drug efflux transporter	Transport			2.13		
PA5206	*argE*	acetylornithine deacetylase	Enzyme					2.22
PA5212		hypothetical protein	Hypothetical		2.24	3.091	2.909	4.636
PA5226		hypothetical protein	Hypothetical			2.88		
PA5270		hypothetical protein	Hypothetical			2.07		
PA5271		hypothetical protein	Hypothetical		3.385	3.356	2.909	5.678
PA5276	*lppL*	lipopeptide	Cell surface structure			2.326		
PA5303		hypothetical protein	Hypothetical			2.217		
PA5312		aldehyde dehydrogenase	Enzyme		2.045	2.907		
PA5313		omega amino acid pyruvate transaminase	Enzyme		2.277	2.703		

PA5314		hypothetical protein	Hypothetical			2.719		2.281
PA5316.1						0.343		
PA5356	*glcC*	DNA-binding transcriptional regulator	Regulator			2.37		
PA5359		hypothetical protein	Hypothetical			2.116	2.339	5.009
PA5365	*phoU*	phosphate uptake regulatory protein	Regulator			2.182	2.864	2.545
PA5366	*pstB*	phosphate transporter ATP-binding protein	Transport			2.877	2.8	2.077
PA5367	*pstA*	phosphate ABC transporter membrane protein	Transport			2.368		
PA5368	*pstC*	phosphate ABC transporter membrane proteinTransport				2.875	3.333	
PA5369	*pstS*	phosphate ABC transporter substrate binding protein	Transport			2.118	3.382	2.882
PA5369.1		5S ribosomal RNA	Ribosome		2.576	3.171		
PA5396		hypothetical protein	Hypothetical			2.087		
PA5404		hypothetical protein	Hypothetical			2.241		
PA5409		hypothetical protein	Hypothetical					2.878
PA5424		hypothetical protein	Hypothetical			2.25		2.828
PA5445		coenzyme A transferase	Enzyme			2.264		3.604
PA5446		hypothetical protein	Hypothetical			3.932		
PA5460		hypothetical protein	Hypothetical			2.7		4
PA5461		hypothetical protein	Hypothetical			2.264		
PA5465		hypothetical protein	Hypothetical					2.343
PA5473		hypothetical protein	Hypothetical			2.19		
PA5475		hypothetical protein	Hypothetical					2.95
PA5481		hypothetical protein	Hypothetical			4.039	13.447	21.368
PA5482		hypothetical protein	Hypothetical			3.346	16.52	14.646
PA5494		hypothetical protein	Hypothetical		2.406	3.131		2.442
PA5516	*pdxY*	pyridoxamine kinase	Enzyme			2.031		
PA5523		aminotransferase	Enzyme		2.257	2.118		
PA5530		MFS dicarboxylate transporter	Transport				2.753	3.507

[a] Fold change of genes at indicated time points compared to 5 min sample, where a fold change of 1 indicates no difference. Green shading indicates genes with elevated RNA levels compared to 5 min (fold change > 1). Red shading indicates genes with reduced RNA levels compared to 5 min (fold change < 1). Grey shading indicates no sigicant difference in gene expression compared to 5 min.

Table S2: Surface-specific gene regulation.

PA number	Gene name	Gene product	Fold expression difference (30 min [surface] vs 5 min post-attachment [a])		
			Plastic [b]	Silicone	Glass
PA0005	*lptA*	lysophosphatidic acid acyltransferase, LptA	0.541		
PA0006	-	D,D-heptose 1,7-bisphosphate phosphatase	0.572		
PA0007	-	hypothetical protein		0.310	
PA0020	-	hypothetical protein	0.302		
PA0024	*hemF*	coproporphyrinogen III oxidase	0.421		0.354
PA0026	*plcB*	phospholipase C, PlcB	0.462		
PA0036	*trpB*	tryptophan synthase subunit beta		7.619	
PA0038	-	hypothetical protein		0.346	
PA0045	-	hypothetical protein		2.344	
PA0046	-	hypothetical protein		4.616	
PA0047	-	hypothetical protein		2.158	
PA0050	-	hypothetical protein	0.606		
PA0055	-	hypothetical protein	0.583		
PA0059	*osmC*	osmotically inducible protein OsmC		0.199	
PA0061	-	hypothetical protein			1.938
PA0094	-	hypothetical protein	0.639		
PA0109	-	hypothetical protein	3.824	0.313	
PA0122	-	hypothetical protein		0.290	
PA0124	-	hypothetical protein		2.045	
PA0125	-	hypothetical protein		2.069	
PA0130	-	aldehyde dehydrogenase	2.604	0.429	
PA0131	-	hypothetical protein	2.582	0.426	
PA0165	-	hypothetical protein		2.345	
PA0170	-	hypothetical protein		4.444	
PA0171	-	hypothetical protein		4.333	
PA0172	-	hypothetical protein		2.704	
PA0176	*aer2*	aerotaxis transducer Aer2		0.194	
PA0177	-	purine-binding chemotaxis protein	2.600	0.217	
PA0178	-	two-component sensor		0.183	
PA0179	-	two-component response regulator	2.771	0.150	
PA0200	-	hypothetical protein	3.462		
PA0201	-	hypothetical protein	0.251	3.460	
PA0250	-	hypothetical protein		0.455	
PA0253	-	transcriptional regulator		3.100	
PA0256	-	hypothetical protein		0.321	
PA0263	*hcpC*	secreted protein Hcp		3.524	
PA0263.1	-	Arg tRNA			
PA0276	-	hypothetical protein			5.333
PA0277	-	hypothetical protein		2.321	
PA0283	*sbp*	sulfate-binding protein		9.438	
PA0284	-	hypothetical protein		19.977	
PA0291	*oprE*	anaerobically-induced outer membrane porin OprE	0.558		
PA0293	*aguB*	N-carbamoylputrescine amidohydrolase	2.833		
PA0297	*spuA*	glutamine amidotransferase			
PA0307	-	hypothetical proteinhypothetical protein	0.278		0.378

PA0329	-	hypothetical protein		0.460	
PA0330	*rpiA*	ribose-5-phosphate isomerase A	0.665		
PA0332	-	hypothetical protein		0.406	
PA0338	-	hypothetical protein	0.638		
PA0341	*lgt*	prolipoprotein diacylglyceryl transferase		2.455	
PA0342	*thyA*	thymidylate synthase	0.620		
PA0346	-	hypothetical protein	0.589		
PA0355	*pfpI*	protease PfpI		0.167	
PA0359	-	hypothetical protein			0.385
PA0363	*coaD*	phosphopantetheine adenylyltransferase	0.594		
PA0385	-	hypothetical protein	0.674		
PA0395	*pilT*	twitching motility protein PilT	0.642		
PA0396	*pilU*	twitching motility protein PilU	0.583		
PA0398	-	hypothetical protein	0.675		
PA0407	*gshB*	glutathione synthetase	0.589		
PA0420	*bioA*	adenosylmethionine-8-amino-7-oxononanoate aminotransferase	0.541		
PA0447	*gcdH*	glutaryl-CoA dehydrogenase		0.311	
PA0449	-	hypothetical protein		0.444	
PA0459	-	ClpA/B protease ATP binding subunit		0.490	
PA0460	-	hypothetical protein		0.388	
PA0462	-	hypothetical protein		0.432	
PA0483	-	acetyltransferase		0.417	
PA0484	-	hypothetical protein		0.263	
PA0490	-	hypothetical protein		0.462	
PA0505	-	hypothetical protein		0.444	
PA0506	-	acyl-CoA dehydrogenase	0.495		
PA0508	-	acyl-CoA dehydrogenase		5.944	
PA0509	*nirN*	cytochrome C		0.268	
PA0510	-	uroporphyrin-III C-methyltransferase		0.203	
PA0511	*nirJ*	heme d1 biosynthesis protein NirJ		0.252	
PA0512	-	hypothetical protein		0.443	
PA0513	-	transcriptional regulator		0.269	
PA0515	-	transcriptional regulator		0.369	
PA0516	*nirF*	heme d1 biosynthesis protein NirF		0.402	
PA0517	*nirC*	cytochrome C		0.388	
PA0518	*nirM*	cytochrome C-551		0.387	
PA0519	*nirS*	nitrite reductase		0.262	
PA0520	*nirQ*	regulatory protein NirQ	0.402	0.261	
PA0523	*norC*	nitric-oxide reductase subunit C	0.317	0.134	
PA0524	*norB*	nitric-oxide reductase subunit B		0.239	
PA0526	-	hypothetical protein		0.151	
PA0527.1	*rsmY*	-	0.124		
PA0529	-	hypothetical protein	2.407		2.000
PA0530	-	class III pyridoxal phosphate-dependent aminotransferase			4.364
PA0531	-	glutamine amidotransferase			3.833
PA0535	-	transcriptional regulator			2.171
PA0541	-	hypothetical protein	0.559		
PA0553	-	hypothetical protein		0.473	
PA0563	-	hypothetical protein	0.516		
PA0567	--	hypothetical protein	0.684	0.200	0.411
PA0585	-	hypothetical protein	2.542		

PA0586	-	SpoVR family protein		0.204	
PA0587	-	hypothetical protein		0.289	
PA0588	-	hypothetical protein		0.283	
PA0592	*ksgA*	dimethyladenosine transferase	0.621		
PA0601	-	two-component response regulator			2.444
PA0602	-	ABC transporter	0.302		
PA0603	-	ABC transporter ATP-binding protein		3.211	
PA0612	*ptrB*	repressor PtrB			3.065
PA0613	-	hypothetical protein		0.434	2.798
PA0614	-	hypothetical protein		0.549	3.305
PA0615	-	hypothetical protein			2.538
PA0616	-	hypothetical protein			3.020
PA0617	-	bacteriophage protein		0.367	3.519
PA0618	-	bacteriophage protein			3.220
PA0619	-	bacteriophage protein			2.960
PA0620	-	bacteriophage protein		0.333	
PA0621	-	hypothetical protein		0.421	
PA0622	-	bacteriophage protein		0.248	2.584
PA0623	--	bacteriophage protein	0.676	0.214	2.048
PA0624	-	hypothetical protein			4.043
PA0625	-	hypothetical protein		0.264	2.824
PA0626	-	hypothetical protein			2.385
PA0627	-	hypothetical protein			2.619
PA0628	-	hypothetical protein		0.232	2.415
PA0629	-	hypothetical protein	0.564	0.167	
PA0631	-	hypothetical protein			3.688
PA0633	-	hypothetical protein		0.373	
PA0634	-	hypothetical protein		0.453	3.608
PA0635	-	hypothetical protein			3.392
PA0636	-	hypothetical protein			2.718
PA0637	-	hypothetical protein			2.367
PA0638	-	bacteriophage protein			3.409
PA0639	-	hypothetical protein			4.540
PA0640	-	bacteriophage protein	0.677	0.258	
PA0641	-	bacteriophage protein			2.654
PA0643	-	hypothetical protein			2.159
PA0644	-	hypothetical protein		0.000	
PA0647	-	hypothetical protein		0.005	2.262
PA0648	-	hypothetical protein		0.040	
PA0654	*speD*	S-adenosylmethionine decarboxylase		0.014	
PA0656	-	HIT family protein		0.000	
PA0659	-	hypothetical protein	0.540		
PA0663	-	hypothetical protein	0.569		
PA0665	-	iron-sulfur cluster insertion protein ErpA	0.642		
PA0668.1	-	16S ribosomal RNA		0.000	
PA0668.3	-	Ala tRNA		0.028	
PA0668.4	-	23S ribosomal RNA		0.000	
PA0668.5	-	5S ribosomal RNA	0.467	0.001	
PA0712	-	hypothetical protein	0.524		
PA0714.1	*phrD*	-	0.356		0.332
PA0729.1	-	Gly tRNA	0.623		
PA0730	-	transferase		0.005	
PA0734	-	hypothetical protein	0.558		
PA0736a	-	hypothetical protein		0.000	

PA0745	-	enoyl-CoA hydratase		0.000	
PA0760	-	hypothetical protein	0.630		
PA0764	*mucB*	negative regulator for alginate biosynthesis MucB		0.010	
PA0766	*mucD*	serine protease MucD	0.410		
PA0788	-	hypothetical protein	5.900		2.650
PA0788a	-	hypothetical protein	2.347		
PA0798	*pmtA*	phospholipid methyltransferase	0.537	0.000	
PA0807	*ampDh3*	protein AmpDh3			2.417
PA0830	-	hypothetical protein			4.170
PA0839	--	transcriptional regulator	0.590	0.000	0.252
PA0849	*trxB2*	thioredoxin reductase	3.043		
PA0852	*cbpD*	chitin-binding protein CbpD	0.676		
PA0867	*mliC*	lysozyme inhibitor		0.004	
PA0870	*phhC*	aromatic amino acid aminotransferase		0.000	
PA0887	*acsA*	acetyl-CoA synthetase		0.019	
PA0899	*aruB*	succinylarginine dihydrolase		0.012	
PA0905.3	-	Arg tRNA	0.490		
PA0907	-	hypothetical protein			3.211
PA0910	-	hypothetical protein			2.813
PA0911	-	hypothetical protein			3.217
PA0915	-	hypothetical protein		0.000	
PA0916	-	hypothetical protein		0.030	
PA0918	-	cytochrome b561		0.000	
PA0921	-	hypothetical protein	0.415		
PA0922.1	-	Met tRNA	0.417		
PA0929	-	two-component response regulator		0.001	
PA0936	*lpxO2*	lipopolysaccharide biosynthetic protein LpxO2	0.680		
PA0941	-	hypothetical protein		0.001	
PA0952	-	hypothetical protein	0.266	0.006	
PA0960	-	hypothetical protein		0.001	
PA0961	-	cold-shock protein	0.500		
PA0975	-	radical activating enzyme		0.046	
PA0976.1	-	Lys tRNA	0.245		
PA0982	-	hypothetical protein		0.001	
PA0983	-	hypothetical protein	2.661	0.005	
PA0984	-	colicin immunity protein			3.049
PA0988	-	hypothetical protein	0.568		
PA0996	*pqsA*	coenzyme A ligase	0.262	0.000	0.274
PA0997	*pqsB*	PqsB protein	0.393	0.000	
PA0999	*pqsD*	3-oxoacyl-ACP synthase	0.610	0.000	
PA1000	*pqsE*	quinolone signal response protein	0.674	0.000	
PA1001	*phnA*	anthranilate synthase component I		0.045	
PA1009	-	hypothetical protein	0.609		
PA1010	*dapA*	dihydrodipicolinate synthase	0.509		
PA1013.1	-	Ser tRNA	0.519	0.000	
PA1026	-	hypothetical protein		2.113	
PA1030.1	-	-			1.865
PA1034	-	hypothetical protein	0.369		
PA1035	-	hypothetical protein	0.569		
PA1041	-	hypothetical protein		0.242	
PA1042	-	hypothetical protein	0.604		
PA1051	-	transporter		0.482	

PA1082	*flgG*	flagellar basal body rod protein FlgG	0.635	0.516	
PA1098	*fleS*	two-component sensor	0.590	0.458	
PA1100	*fliE*	flagellar hook-basal body protein FliE		0.539	
PA1118	-	hypothetical protein		0.566	
PA1123	-	hypothetical protein	0.593		
PA1126	-	hypothetical protein	0.600		
PA1159	-	cold-shock protein	0.505		
PA1172	*napC*	cytochrome C protein NapC	2.738	0.254	
PA1173	*napB*	cytochrome C protein NapB	2.500	0.255	
PA1175	*napD*	NapD protein of periplasmic nitrate reductase		0.223	
PA1176	*napF*	ferredoxin protein NapF	2.278		
PA1177	*napE*	periplasmic nitrate reductase protein NapE		0.130	
PA1179	*phoP*	two-component response regulator PhoP	0.553		0.350
PA1180	*phoQ*	two-component sensor PhoQ	0.584		0.310
PA1183	*dctA*	C4-dicarboxylate transporter DctA		2.387	
PA1191	-	hypothetical protein		0.444	
PA1197	-	NAD-dependent deacetylase	3.000		
PA1244	-	hypothetical protein		0.465	
PA1248	*aprF*	alkaline protease secretion outer membrane protein AprF	0.351	0.321	
PA1249	*aprA*	alkaline metalloproteinase	0.550	0.125	
PA1250	*aprI*	alkaline proteinase inhibitor AprI	0.525		
PA1272	*cobO*	cob(I)yrinic acid a,c-diamide adenosyltransferase		2.098	
PA1289	-	hypothetical protein	3.974		
PA1295	-	hypothetical protein		2.980	
PA1299	-	hypothetical protein		1.963	
PA1323	-	hypothetical protein		0.424	
PA1324	-	hypothetical protein		0.226	
PA1333	-	hypothetical protein	2.170	0.500	
PA1337	*ansB*	glutaminase-asparaginase		0.333	
PA1343	-	hypothetical protein	0.123	3.357	
PA1344	-	short-chain dehydrogenase		3.086	
PA1358	-	hypothetical protein		0.408	
PA1366	-	hypothetical protein		0.455	
PA1404	-	hypothetical protein	0.630	0.502	
PA1420	-	hypothetical protein	3.043		
PA1442	-	flagellar basal body protein FliL	0.648		
PA1452	*flhA*	flagellar biosynthesis protein FlhA	0.628		
PA1459	-	chemotaxis-specific methylesterase		0.475	
PA1460	*motC*	flagellar motor protein		0.374	
PA1461	*motD*	flagellar motor protein MotD		0.464	
PA1471	-	hypothetical protein		0.355	
PA1473	-	hypothetical protein	2.592		
PA1478	-	hypothetical protein	0.682		
PA1479	*ccmE*	cytochrome C biogenesis protein CcmE	0.683		
PA1481	*ccmG*	cytochrome C biogenesis protein CcmG	0.659		
PA1482	*ccmH*	cytochrome C biogenesis protein CcmH	0.653		
PA1512	*hcpA*	secreted protein Hcp		3.445	
PA1520	-	transcriptional regulator	0.682		
PA1524	*xdhA*	xanthine dehydrogenase	3.737		

PA1530	-	hypothetical protein	0.693		
PA1543	*apt*	adenine phosphoribosyltransferase	0.616		
PA1561	*aer*	aerotaxis receptor Aeraerotaxis receptor Aer	4.244	0.488	
PA1562	*acnA*	aconitate hydratase		0.240	
PA1571	-	hypothetical protein	0.612		
PA1573	-	hypothetical protein	2.324		
PA1576	-	3-hydroxyisobutyrate dehydrogenase	0.550		
PA1578a	-	hypothetical protein		2.102	
PA1590	*braB*	branched-chain amino acid transporter	0.678		
PA1604	-	hypothetical protein	3.461		
PA1630	-	transcriptional regulator		2.044	
PA1643a	-	aldehyde-activating protein	2.736		
PA1653	-	transcriptional regulator	0.632		
PA1662	-	ClpA/B-type protease	2.778		
PA1663	-	transcriptional regulator	5.067		2.933
PA1664	-	hypothetical protein	7.958	2.667	
PA1665	-	hypothetical protein	3.550	2.250	
PA1666	-	hypothetical protein	3.667		2.359
PA1667	-	hypothetical protein	2.344		
PA1668	-	hypothetical protein		2.444	2.556
PA1670	*stp1*	serine/threonine phosphoprotein phosphatase Stp1			5.182
PA1677	-	hypothetical protein	0.474		
PA1683	-	methylthioribulose-1-phosphate dehydratase	0.637		
PA1687	*speE*	spermidine synthase		2.182	
PA1688	--	hypothetical protein		3.057	4.243
PA1689	-	hypothetical protein		2.579	2.368
PA1713	*exsA*	transcriptional regulator ExsA	0.390		
PA1718	*pscE*	type III export protein PscE		3.588	
PA1719	*pscF*	type III export protein PscF		3.000	
PA1728	-	hypothetical protein		0.436	
PA1745	-	hypothetical protein	2.541	0.306	
PA1746	-	hypothetical protein		0.431	
PA1753	-	hypothetical protein		0.420	
PA1757	*thrH*	phosphoserine phosphatase	0.571		
PA1772	-	ribonuclease activity regulator protein RraA	0.705		
PA1788	-	hypothetical protein	0.687		
PA1789	-	hypothetical protein	2.867		
PA1791	-	hypothetical protein		2.324	
PA1796.1	-	Arg tRNA	0.526		
PA1796.4	-	His tRNA	0.620		
PA1806	*fabI*	NADH-dependent enoyl-ACP reductase		0.519	
PA1828	-	short-chain dehydrogenaseshort-chain dehydrogenase	2.377		3.528
PA1831	-	hypothetical protein	0.409		
PA1832	-	periplasmic protease	2.567		
PA1837a	-	hypothetical protein	3.367	0.463	
PA1838	*cysI*	sulfite reductase		2.154	
PA1839	-	ribosomal RNA large subunit methyltransferase N		2.100	
PA1852	-	hypothetical protein			

PA1853	-	transcriptional regulator	0.457		
PA1860	-	hypothetical proteinhypothetical protein		0.208	
PA1869	-	acyl carrier proteinacyl carrier protein	0.191		
PA1887	-	hypothetical protein	4.235		
PA1889	-	hypothetical protein	0.438		
PA1930	-	chemotaxis transducer	3.173	0.259	
PA1942	-	hypothetical protein		0.443	
PA1944	-	hypothetical protein	2.286		
PA1946	*rbsB*	ribose ABC transporter substrate-binding protein		0.384	
PA1950	*rbsK*	ribokinaseribokinase	0.602	0.466	
PA1966	-	hypothetical protein		1.880	
PA1970	-	hypothetical protein		0.522	
PA1985	*pqqA*	coenzyme PQQ synthesis protein PqqA	0.659	0.519	0.317
PA1999	*dhcA*	dehydrocarnitine CoA transferase subunit A	0.439	0.497	
PA2007	*maiA*	maleylacetoacetate isomerase		0.161	
PA2009	*hmgA*	homogentisate 1,2-dioxygenase		0.224	
PA2011	*liuE*	hydroxymethylglutaryl-CoA lyase	0.697	0.182	
PA2015	*liuA*	isovaleryl-CoA dehydrogenase		0.283	
PA2016	*liuR*	regulator of liu genes		0.321	
PA2021	-	hypothetical protein		0.504	
PA2024	-	ring-cleaving dioxygenase	2.971		
PA2045	-	hypothetical protein	0.556		0.374
PA2072	-	hypothetical protein	2.314		
PA2118a	-	hypothetical protein	3.417		3.268
PA2142a	-	hypothetical protein	3.310	0.327	
PA2143	-	hypothetical protein		0.148	
PA2166	-	hypothetical protein	2.171		2.300
PA2171	-	hypothetical protein		0.000	0.364
PA2174	-	hypothetical protein		0.000	
PA2190	-	hypothetical protein		0.000	
PA2193	*hcnA*	hydrogen cyanide synthase HcnA	0.406		
PA2199	-	dehydrogenase	0.683		
PA2204	-	ABC transporter		0.000	
PA2223	-	hypothetical protein		0.001	
PA2224	-	hypothetical protein		0.010	
PA2231	*pslA*	protein PslA		0.013	
PA2235	*pslE*	protein PslE		0.037	
PA2246	*bkdR*	transcriptional regulator BkdR	0.495		
PA2250	*lpdV*	dihydrolipoamide dehydrogenase	0.388	0.000	
PA2282	-	hypothetical protein			4.091
PA2365	-	hypothetical protein		0.000	
PA2366	-	uricase	2.576		
PA2367	-	hypothetical protein	2.396		
PA2373	-	hypothetical protein		0.000	
PA2380	-	hypothetical protein	0.521		
PA2398	*fpvA*	ferripyoverdine receptor		0.005	
PA2433	-	hypothetical protein		0.000	
PA2436	-	hypothetical protein	0.498		
PA2457	-	hypothetical protein		0.023	
PA2460	-	hypothetical protein	0.623		
PA2485	-	hypothetical protein		0.003	
PA2486	-	hypothetical protein	2.465	0.000	

PA2490	-	hypothetical protein	0.572		
PA2504	-	hypothetical protein	2.179		
PA2532	*tpx*	thiol peroxidase		0.009	
PA2533	-	sodium:alanine symporter	0.569		
PA2541	-	CDP-alcohol phosphatidyltransferase		0.012	
PA2550	-	acyl-CoA dehydrogenase			2.205
PA2554	-	short-chain dehydrogenase		0.000	
PA2555	-	AMP-binding protein		0.000	
PA2562	-	hypothetical protein		0.000	
PA2564	-	hypothetical protein	2.517		2.310
PA2569	-	hypothetical protein	0.687		
PA2570	*lecA*	LecA protein		0.002	
PA2571	-	two-component sensor		0.000	
PA2572	-	two-component response regulator	2.464	0.000	
PA2573	-	chemotaxis transducer		0.019	
PA2581.1	-	Cys tRNA	0.419		
PA2618	-	arginyl-tRNA-protein transferase		0.000	
PA2619	*infA*	translation initiation factor IF-1	0.451		
PA2622	*cspD*	cold-shock protein CspD		0.039	
PA2632	-	hypothetical protein	2.783		
PA2637	*nuoA*	NADH dehydrogenase subunit A	0.523		
PA2638	*nuoB*	NADH dehydrogenase subunit B	0.582		
PA2644	*nuoI*	NADH dehydrogenase subunit I			1.964
PA2645	*nuoJ*	NADH dehydrogenase subunit J			2.120
PA2652	-	chemotaxis transducer			2.189
PA2653	-	transporter		0.000	
PA2655	-	hypothetical protein	0.679	0.000	
PA2663	*ppyR*	psl and pyoverdine operon regulator, PpyR	0.669	0.000	0.218
PA2664	*fhp*	nitric oxide dioxygenase	0.164	0.000	0.059
PA2685	-	hypothetical protein	0.655		
PA2706	-	hypothetical protein			1.909
PA2713	-	hypothetical protein			2.895
PA2720	-	hypothetical protein	0.630		
PA2736.1	-	Pro tRNAPro tRNA	0.460	0.013	
PA2746a	-	hypothetical protein	3.975		
PA2747	-	hypothetical protein	4.138		4.292
PA2750a	-	hypothetical protein	0.575		
PA2753	-	hypothetical protein	3.264	0.001	
PA2754	-	hypothetical protein		0.002	
PA2755a	-	hypothetical protein		0.331	
PA2759	--	hypothetical protein	4.592	0.398	3.766
PA2769	-	hypothetical protein			2.087
PA2770	-	hypothetical protein		2.132	
PA2772	-	hypothetical protein		2.319	
PA2775.1	-	Val tRNA		5.455	
PA2779	-	hypothetical protein	2.605	0.462	
PA2788	-	chemotaxis transducer		0.329	
PA2795	-	tRNA-dihydrouridine synthase A	0.602		
PA2812	-	ABC transporter ATP-binding protein	0.570		
PA2817	-	hypothetical protein		2.721	
PA2819.1	-	Gly tRNA	0.416		
PA2852.1	-	Ser tRNASer tRNA	0.159		
PA2856	*tesA*	acyl-CoA thioesterase	0.659		

PA2860	-	hypothetical protein	0.277		
PA2866	*mttC*	secretion protein MttC	0.687		
PA2870	-	hypothetical protein	2.619		
PA2880	-	hypothetical protein		5.000	
PA2896	-	RNA polymerase sigma factor		0.523	
PA2902	-	hypothetical protein	0.455		
PA2920	-	chemotaxis transducer	2.667		
PA2937	-	hypothetical protein	3.112		
PA2939	-	aminopeptidase		0.275	
PA2953	-	electron transfer flavoprotein-ubiquinone oxidoreductase	0.486		
PA2957	-	transcriptional regulator		2.347	
PA2969	*plsX*	glycerol-3-phosphate acyltransferase PlsX	0.682		
PA2973	-	peptidase	0.656		
PA2974	-	hydrolase	0.468		
PA2983	-	tolQ-type transporter	0.600		
PA2985	-	hypothetical protein	0.639		
PA2986	-	hypothetical protein	0.626		
PA2988	-	hypothetical protein	0.556		
PA3004	-	5'-methylthioadenosine phosphorylase	0.635		
PA3006	*psrA*	transcriptional regulator PsrA		2.296	3.071
PA3009	-	hypothetical protein	0.497		
PA3012	-	hypothetical protein	0.632		
PA3013	*foaB*	3-ketoacyl-CoA thiolase	0.406		
PA3014	*faoA*	multifunctional fatty acid oxidation complex subunit alpha	0.329		
PA3017	-	hypothetical protein			2.500
PA3018	-	hypothetical protein		2.070	
PA3040	-	hypothetical protein		0.275	
PA3041	-	hypothetical protein		0.336	
PA3049	*rmf*	ribosome modulation factor		0.185	
PA3069	-	hypothetical protein		0.449	
PA3091	-	hypothetical protein	0.268		
PA3094.1	-	Asp tRNA	0.503		
PA3094.3	-	Val tRNA	0.325		
PA3106	-	oxidoreductase	0.536		
PA3109	-	hypothetical protein	0.591		
PA3112	*accD*	acetyl-CoA carboxylase subunit beta	0.508		
PA3116	-	aspartate-semialdehyde dehydrogenase	3.000		
PA3123	-	hypothetical protein		0.253	
PA3126	*ibpA*	heat-shock protein IbpA	0.551	0.282	
PA3133.1	-	Glu tRNA	0.545		
PA3133.2	-	Ala tRNA	0.625		
PA3139	-	aromatic amino acid aminotransferase	0.548		
PA3139.1	-	Asn tRNA	0.451		
PA3142	-	hypothetical protein	0.631	0.460	
PA3171	*ubiG*	3-demethylubiquinone-9 3-methyltransferase	0.670		
PA3179	-	hypothetical protein		3.092	
PA3181	-	keto-hydroxyglutarate-aldolase/keto-deoxy-phosphogluconate aldolase		0.488	
PA3183	*zwf*	glucose-6-phosphate 1-dehydrogenase		0.514	

PA3190	-	sugar ABC transporter substrate-binding protein	0.519		
PA3205	--	hypothetical protein	2.865	2.683	2.746
PA3220	-	transcriptional regulator	0.345		0.361
PA3221	*csaA*	CsaA protein	0.627		
PA3227	*ppiA*	peptidyl-prolyl cis-trans isomerase A		0.246	
PA3229	-	hypothetical protein		0.504	
PA3248	-	hypothetical protein			1.931
PA3262.1	-	Asp tRNA	0.631		
PA3262.2	-	Val tRNA	0.298		
PA3266	*capB*	cold acclimation protein B		3.167	
PA3277	-	short-chain dehydrogenase			2.500
PA3278	--	hypothetical protein	0.639	0.472	
PA3291	-	hypothetical protein		2.357	
PA3295	-	HIT family protein	0.582		
PA3303a	-	hypothetical protein	2.518		
PA3304.1	-	-	2.589		
PA3310	-	hypothetical protein		2.667	2.667
PA3337	*rfaD*	ADP-L-glycero-D-manno-heptose-6-epimerase	3.093		
PA3341	-	transcriptional regulatortranscriptional regulator	0.669	0.531	
PA3346	-	two-component response regulator		0.436	
PA3347	-	hypothetical protein		0.485	
PA3350	-	flagellar basal body P-ring biosynthesis protein FlgA	0.513		
PA3353	-	hypothetical protein		0.539	
PA3361	*lecB*	fucose-binding lectin PA-IIL	2.516	0.375	
PA3362	-	hypothetical protein		0.480	
PA3363	*amiR*	aliphatic amidase regulator		0.395	
PA3366.1	*amiL*	-			
PA3369	-	hypothetical protein	10.866		3.307
PA3370	-	hypothetical protein	5.750		3.194
PA3385	*amrZ*	alginate and motility regulator Z		0.314	
PA3391	*nosR*	regulatory protein NosR		0.230	
PA3392	*nosZ*	nitrous-oxide reductase		0.414	
PA3396	*nosL*	NosL protein	2.980		
PA3417	-	pyruvate dehydrogenase E1 component subunit alpha	2.542		
PA3418	*ldh*	leucine dehydrogenase		0.159	
PA3430	-	aldolase			2.857
PA3432	-	hypothetical protein	5.037		
PA3450	-	antioxidant protein		9.547	
PA3451	-	hypothetical protein		0.307	
PA3456	-	5-methylaminomethyl-2-thiouridine methyltransferase		2.278	
PA3458	-	transcriptional regulator		0.255	
PA3465	-	hypothetical protein	2.493	0.381	
PA3472	-	hypothetical protein	0.664		
PA3476	*rhlI*	autoinducer synthesis protein RhlI	0.673		
PA3488	-	hypothetical protein		2.186	
PA3496	-	hypothetical protein	0.478		
PA3519	-	hypothetical protein			4.450
PA3520	-	hypothetical protein		0.368	

PA3526	-	hypothetical protein			3.173
PA3552	*arnB*	UDP-4-amino-4-deoxy-L-arabinose-oxoglutarate aminotransferase	0.203	3.235	
PA3553	*arnC*	protein ArnC		5.429	
PA3558	*arnF*	hypothetical protein			0.364
PA3570	*mmsA*	methylmalonate-semialdehyde dehydrogenase	0.632		
PA3572	-	hypothetical protein	4.259	0.443	
PA3574a	-	copper chaperone CopZ		2.426	2.715
PA3578	-	hypothetical protein	0.702		
PA3580	-	hypothetical protein	0.698		
PA3584	*glpD*	glycerol-3-phosphate dehydrogenase		0.407	
PA3601	-	50S ribosomal protein L31			0.349
PA3614	-	hypothetical protein	3.912		
PA3615	-	hypothetical protein	2.827		
PA3621.1	*rsmZ*	-	0.223		
PA3626	-	tRNA pseudouridine synthase D	0.675		
PA3629	*adhC*	alcohol dehydrogenase		0.384	
PA3634	-	hypothetical protein			2.635
PA3636	*kdsA*	2-dehydro-3-deoxyphosphooctonate aldolase	0.643		
PA3639	*accA*	acetyl-CoA carboxylase carboxyltransferase subunit alpha	0.471		
PA3645	*fabZ*	(3R)-hydroxymyristoyl-ACP dehydratase	0.544		
PA3649	-	hypothetical protein	0.622		
PA3655	*tsf*	elongation factor Ts		2.740	
PA3663	-	hypothetical protein	0.637	2.368	
PA3664	-	hypothetical protein	0.476		
PA3673	*plsB*	glycerol-3-phosphate acyltransferase	0.455		
PA3684	-	hypothetical protein		0.434	
PA3685	-	hypothetical protein	0.560		
PA3691	-	hypothetical protein		0.277	
PA3692	*lptF*	lipotoxon F LptF		0.197	
PA3694	-	hypothetical protein	0.580		
PA3703	*wspF*	chemotaxis-specific methylesterase		0.553	
PA3712	-	hypothetical protein		0.373	
PA3717	-	FkbP-type peptidyl-prolyl cis-trans isomerase	0.648		
PA3720	-	hypothetical protein		3.545	
PA3721	*nalC*	NalC protein		3.667	
PA3722	-	hypothetical protein	0.478		
PA3723	-	FMN oxidoreductase	2.976		
PA3724	*lasB*	elastase LasB	0.576		
PA3727	-	hypothetical protein		0.474	
PA3762	-	hypothetical protein	0.544	0.549	
PA3767	-	hypothetical protein	3.440		
PA3791	-	hypothetical protein	0.559		
PA3812	*iscA*	iron-binding protein IscA			2.015
PA3823	*tgt*	queuine tRNA-ribosyltransferase	0.441		
PA3824	*queA*	S-adenosylmethionine-tRNA ribosyltransferase-isomerase	0.669		
PA3824.1	-	Leu tRNA	0.434		
PA3827	-	hypothetical protein	0.688		
PA3846	-	hypothetical protein			2.708

PA3858	-	amino acid-binding protein		0.409	
PA3870	*moaA1*	molybdenum cofactor biosynthesis protein A	15.286		8.000
PA3871	--	PpiC-type peptidyl-prolyl cis-trans isomerase	23.313	2.875	15.000
PA3872	*narI*	respiratory nitrate reductase subunit gamma	23.238	2.524	13.333
PA3873	*narJ*	respiratory nitrate reductase subunit delta	20.400		18.200
PA3874	*narH*	respiratory nitrate reductase subunit beta	16.250		9.893
PA3875	*narG*	respiratory nitrate reductase subunit alpha	6.596		3.532
PA3876	*narK2*	nitrite extrusion protein 2	0.595	0.027	
PA3877	*narK1*	nitrite extrusion protein 1	0.146	0.013	0.123
PA3878	*narX*	two-component sensor NarX			2.000
PA3879	*narL*	transcriptional regulator NarL			2.382
PA3880	-	hypothetical protein		0.490	
PA3911	-	hypothetical protein	2.235		
PA3913	-	protease	0.663		
PA3914	*moeA1*	molybdenum cofactor biosynthetic protein A1	0.269	0.019	0.298
PA3915	*moaB1*	molybdopterin biosynthetic protein B1	0.092	0.014	0.070
PA3916	*moaE*	molybdopterin converting factor large subunit	0.234	0.127	0.243
PA3917	*moaD*	molybdopterin converting factor small subunit	0.282	0.167	0.211
PA3918	*moaC*	molybdenum cofactor biosynthesis protein MoaC	0.230	0.108	0.225
PA3919	-	hypothetical protein		0.378	
PA3945	-	hypothetical protein		0.308	
PA3966	-	hypothetical protein	0.633		
PA3967	-	hypothetical protein	0.495		
PA3973	-	transcriptional regulator			2.083
PA3986	-	hypothetical protein		0.225	
PA3997	*lipB*	lipoate-protein ligase B	0.574		
PA4003	*pbpA*	penicillin-binding protein 2	0.653		
PA4006	*nadD*	nicotinic acid mononucleotide adenylyltransferase	0.672		
PA4010	-	3-methyladenine DNA glycosylase	0.416		
PA4012	-	hypothetical protein		0.435	
PA4015	-	hypothetical protein		0.355	
PA4017	-	hypothetical protein		0.538	
PA4020	*mpl*	UDP-N-acetylmuramate:L-alanyl-gamma-D-glutamyl-meso-diaminopimelate ligase	0.561		
PA4027	-	hypothetical protein	4.000		2.300
PA4032	-	two-component response regulator			2.222
PA4042	*xseB*	exodeoxyribonuclease VII small subunit	0.620		
PA4057	*nrdR*	transcriptional regulator NrdR	0.514		
PA4108a	-	hypothetical protein		0.500	
PA4114	-	spermidine acetyltransferase	0.632		
PA4118	-	hypothetical protein	0.570		
PA4131	-	iron-sulfur protein	0.520		

PA4135	-	transcriptional regulator	0.538		
PA4178	-	hypothetical protein		2.286	
PA4236	*katA*	catalase		0.377	
PA4242	*rpmJ*	50S ribosomal protein L36	2.530		
PA4244	*rplO*	50S ribosomal protein L15			2.881
PA4250	*rpsN*	30S ribosomal protein S14		3.707	
PA4251	*rplE*	50S ribosomal protein L5		3.793	
PA4252	*rplX*	50S ribosomal protein L24	2.498	3.554	
PA4254	*rpsQ*	30S ribosomal protein S17		2.484	
PA4269	*rpoC*	DNA-directed RNA polymerase subunit beta'	2.645	3.790	
PA4270.1	--	--	2.201	2.007	2.716
PA4271	*rplL*	50S ribosomal protein L7/L12		4.032	
PA4272	*rplJ*	50S ribosomal protein L10		2.771	
PA4280	*birA*	biotin-protein ligase		2.905	
PA4280.1	-	5S ribosomal RNA	0.469	0.481	
PA4280.2	-	23S ribosomal RNA		0.080	
PA4280.3	-	Ala tRNA		3.173	
PA4280.5	-	16S ribosomal RNA		0.128	
PA4294	-	hypothetical protein	3.000		
PA4296	*pprB*	two-component response regulator, PprB	3.911	0.362	
PA4305	*rcpC*	RcpC protein			2.520
PA4306	*flp*	type IVb pilin Flp		0.288	
PA4309	*pctA*	chemotactic transducer PctA		0.427	
PA4319	-	hypothetical protein	0.642		
PA4321	-	hypothetical protein	0.698		
PA4325	-	hypothetical protein	0.471		
PA4326	-	hypothetical protein			2.410
PA4345	-	hypothetical protein		0.397	
PA4348	-	hypothetical protein			
PA4362	-	hypothetical protein		0.336	
PA4368	-	hypothetical protein			2.037
PA4374	-	resistance-nodulation-cell division (RND) efflux membrane fusion protein	0.343		
PA4376	*pncB2*	nicotinate phosphoribosyltransferase	0.684		
PA4377	-	hypothetical protein		0.496	
PA4381	-	two-component response regulator	0.561		
PA4385	*groEL*	molecular chaperone GroEL		0.325	
PA4386	*groES*	co-chaperonin GroES	0.538	0.327	
PA4389	*speA*	3-ketoacyl-ACP reductase	0.532		
PA4394	-	hypothetical protein	0.653		
PA4404	-	hypothetical protein		2.000	
PA4406.1	-	-	0.566		
PA4411	*murC*	UDP-N-acetylmuramate-L-alanine ligase		0.513	
PA4414	*murD*	UDP-N-acetylmuramoyl-L-alanyl-D-glutamate synthetase		0.463	
PA4415	*mraY*	phospho-N-acetylmuramoyl-pentapeptide-transferase		0.466	
PA4417	*murE*	UDP-N-acetylmuramoylalanyl-D-glutamate-2,6-diaminopimelate ligase		0.488	
PA4421.1	*rnpB*	--	0.181	0.253	
PA4426	-	hypothetical protein	0.665		
PA4440	-	hypothetical protein	0.654		

PA4443	*cysD*	sulfate adenylyltransferase subunit 2		3.709	
PA4452	-	hypothetical protein	0.567		
PA4453	-	hypothetical protein	0.556		
PA4455	-	ABC transporter permease	0.637		
PA4456	-	ABC transporter ATP-binding protein	0.449		
PA4478	-	Maf-like protein	0.574		
PA4487	-	hypothetical protein	2.493		
PA4495	-	hypothetical protein		0.460	
PA4496	-	ABC transporter	0.580		
PA4514	-	outer membrane receptor for iron transport		2.389	
PA4516	-	hypothetical protein	2.222		
PA4517	-	hypothetical protein		3.071	4.429
PA4523	-	hypothetical protein		0.405	
PA4528	*pilD*	type 4 prepilin peptidase PilD	0.641		
PA4530	-	zinc-binding protein	0.607		
PA4531	-	hypothetical protein		0.368	
PA4534	-	hypothetical protein	0.474		
PA4536	-	hypothetical protein	2.633		
PA4539	-	hypothetical protein	0.641		
PA4541.1	-	Lys tRNA	0.485		
PA4541.2	-	Pro tRNA	0.567		
PA4542	*clpB*	ClpB protein	2.902		
PA4548	-	D-amino acid oxidase	0.575		
PA4559	*lspA*	lipoprotein signal peptidase	0.628		
PA4571	-	cytochrome C	2.426		
PA4573	-	hypothetical protein		0.382	2.202
PA4574	-	hypothetical protein		2.033	
PA4577	-	hypothetical protein		0.423	
PA4580	-	hypothetical protein	0.457		
PA4605	-	hypothetical protein		0.510	
PA4607	-	hypothetical protein		0.219	
PA4608	-	hypothetical protein		0.364	
PA4610	-	hypothetical protein		0.239	
PA4611	-	hypothetical protein		0.457	3.713
PA4616	-	c4-dicarboxylate-binding protein		2.939	
PA4623	-	hypothetical protein		0.485	
PA4635a	-	hypothetical protein			2.045
PA4637	-	hypothetical protein			3.762
PA4637a	--	hypothetical protein	0.331	0.209	0.324
PA4639	-	hypothetical protein	0.611		
PA4642	-	hypothetical protein	0.322		0.336
PA4645	-	hypoxanthine-guanine phosphoribosyltransferase		3.215	
PA4648	-	hypothetical protein		0.487	
PA4656	-	hypothetical protein			2.681
PA4658	-	hypothetical protein	2.288		2.273
PA4659	-	transcriptional regulator	2.619		2.952
PA4667	-	hypothetical protein	0.661		
PA4673.1	-	Met tRNA	0.421		
PA4684	-	hypothetical protein	0.644		
PA4690.1	-	5S ribosomal RNA5S ribosomal RNA	0.463	0.481	
PA4690.2	-	23S ribosomal RNA		0.081	
PA4690.3	-	Ala tRNA		3.173	

PA4690.5	-	16S ribosomal RNA		0.128	
PA4698	-	hypothetical protein	0.442		
PA4699	-	hypothetical protein	0.582		
PA4703	-	hypothetical protein		0.217	
PA4704.1	*prrF1*	-	0.280		
PA4704.2	*prrF2*	-	0.335		
PA4715	-	aminotransferase	0.690		
PA4724.1	-	hypothetical protein	0.612		
PA4726.1	-	-		2.018	
PA4726.2	-	-	0.300		
PA4729	*panB*	3-methyl-2-oxobutanoate hydroxymethyltransferase	0.656		
PA4731	*panD*	aspartate alpha-decarboxylase		2.265	
PA4738	-	hypothetical protein		0.363	
PA4741	*rpsO*	30S ribosomal protein S15		4.590	
PA4758.1	-	-	0.448		
PA4759	*dapB*	dihydrodipicolinate reductase	0.636	0.215	
PA4760	*dnaJ*	molecular chaperone DnaJ		0.367	
PA4761	*dnaK*	molecular chaperone DnaK		0.248	
PA4762	*grpE*	heat shock protein GrpE		0.357	
PA4767	-	hypothetical protein		0.557	
PA4773	-	hypothetical protein		3.540	
PA4781	-	cyclic di-GMP phosphodiesterase		0.413	
PA4782	-	hypothetical protein		2.712	
PA4826	-	hypothetical protein		2.504	2.044
PA4840	-	translation initiation factor Sui1		2.308	
PA4853	*fis*	Fis family transcriptional regulator		2.214	
PA4870	-	hypothetical protein	3.158		
PA4874	-	hypothetical protein	2.745		
PA4876	*osmE*	OsmE family transcriptional regulator	0.694	0.215	
PA4877	-	hypothetical protein		0.258	
PA4878	-	transcriptional regulator			2.347
PA4880	-	bacterioferritin		0.177	
PA4907	-	short-chain dehydrogenase	0.588		
PA4915	-	chemotaxis transducer		0.215	
PA4917	-	hypothetical protein		0.430	4.210
PA4918	-	hypothetical protein			2.138
PA4919	*pncB1*	nicotinate phosphoribosyltransferase		0.447	
PA4920	*nadE*	NAD synthetase		0.280	
PA4932	*rplI*	50S ribosomal protein L9		2.495	
PA4933	-	hypothetical protein		3.309	
PA4934	*rpsR*	30S ribosomal protein S18		3.178	
PA4937.2	-	Leu tRNA	0.488		
PA4948	-	hypothetical protein	0.592		
PA4952	-	ribosome-associated GTPase	0.619		
PA4965	-	hypothetical protein	0.661		
PA4968	-	hypothetical protein	0.481		
PA4997	*msbA*	transporter MsbA	0.574		
PA4998	-	hypothetical protein	0.596		
PA5015	*aceE*	pyruvate dehydrogenase subunit E1		0.507	
PA5016	*aceF*	dihydrolipoamide acetyltransferase		0.467	
PA5019	-	hypothetical protein			2.333
PA5024	-	hypothetical protein		10.267	
PA5026	-	hypothetical protein		3.000	2.239

PA5027	-	hypothetical protein	3.795		
PA5030	-	major facilitator superfamily (MFS) transporter	0.452	0.282	
PA5043	*pilN*	type 4 fimbrial biogenesis protein PilN	0.674		
PA5044	*pilM*	type 4 fimbrial biogenesis protein PilM	0.513		
PA5049	*rpmE*	50S ribosomal protein L31	0.531		
PA5062	-	hypothetical protein		3.444	
PA5063	*ubiE*	ubiquinone/menaquinone biosynthesis methyltransferase	0.627		
PA5075	-	ABC transporter permease		2.069	
PA5077	*mdoH*	glucosyltransferase MdoH	0.629		
PA5079	-	D-tyrosyl-tRNA(Tyr) deacylase			2.389
PA5103	-	hypothetical protein		0.510	
PA5105	*hutC*	histidine utilization repressor HutC	2.254		
PA5106	-	N-formimino-L-glutamate deiminase	4.214		
PA5107	*blc*	outer membrane lipoprotein Blc			2.396
PA5108	-	hypothetical protein	0.544	0.567	
PA5117	*typA*	regulatory protein TypA		2.685	
PA5122	-	hypothetical protein	0.662		
PA5133	-	hypothetical protein	0.574		
PA5137	-	hypothetical protein		2.067	
PA5138	-	hypothetical protein		3.450	
PA5139	-	hypothetical protein	0.385	3.171	
PA5160.1	-	Thr tRNA		2.182	
PA5171	*arcA*	arginine deiminase		0.402	
PA5172	*arcB*	ornithine carbamoyltransferase		0.356	
PA5173	*arcC*	carbamate kinase		0.296	
PA5176	-	ADP-ribose diphosphatase NudE	0.608		
PA5178	-	LysM domain/BON superfamily protein	0.484	0.369	
PA5182	-	hypothetical protein	0.479	0.542	
PA5184	-	chorismate mutase	0.579		
PA5194	-	hypothetical protein		2.353	
PA5212	-	hypothetical protein		0.244	
PA5215	*gcvT1*	glycine cleavage system aminomethyltransferase T	0.421		
PA5227.1	*ssrS*	-	0.457		
PA5240	*trxA*	thioredoxin	0.573		
PA5244	-	hypothetical protein	0.418		
PA5252a	-	hypothetical protein			2.288
PA5259	*hemD*	uroporphyrinogen-III synthase			2.355
PA5261	*algR*	alginate biosynthesis regulatory protein AlgR		0.424	
PA5267	*hcpB*	secreted protein Hcp		3.525	2.321
PA5268	*corA*	magnesium/cobalt transporter	0.434		
PA5274	*rnk*	nucleoside diphosphate kinase regulator		2.323	
PA5286	-	hypothetical protein			2.537
PA5289	-	hypothetical protein	0.674		
PA5301	-	transcriptional regulator	0.576		
PA5302	*dadX*	alanine racemase		2.088	
PA5304	*dadA*	D-amino acid dehydrogenase small subunit	2.581		
PA5308	*lrp*	leucine-responsive regulatory protein	0.558		
PA5314	-	hypothetical protein	0.554		

PA5316.1	-	-	0.496		
PA5334	*rph*	ribonuclease PH	0.603		
PA5347	-	hypothetical protein	0.541		
PA5348	-	DNA-binding protein	0.569		
PA5351	*rubA1*	rubredoxin	0.397		
PA5352	-	hypothetical protein	4.941		3.324
PA5353	*glcF*	glycolate oxidase iron-sulfur subunit	7.286		3.667
PA5355	*glcD*	glycolate oxidase subunit GlcD	3.000	0.225	
PA5359	-	hypothetical protein		0.235	
PA5365	*phoU*	phosphate uptake regulatory protein PhoU		2.746	2.051
PA5366	*pstB*	phosphate transporter ATP-binding protein		3.210	2.032
PA5367	*pstA*	phosphate ABC transporter membrane protein		3.533	3.200
PA5369	*pstS*	phosphate ABC transporter substrate-binding protein	3.353	2.294	3.529
PA5369.1	-	5S ribosomal RNA	0.469	0.481	
PA5369.2	-	23S ribosomal RNA		0.081	
PA5369.3	-	Ala tRNA		3.173	
PA5369.5	-	16S ribosomal RNA		0.129	
PA5372	*betA*	choline dehydrogenase	2.698		
PA5381	-	hypothetical protein			
PA5406	-	hypothetical protein		4.158	
PA5407	-	hypothetical protein		2.711	
PA5408	-	hypothetical protein	2.855		2.161
PA5424	-	hypothetical protein	0.584	0.432	
PA5427	*adhA*	alcohol dehydrogenase	2.703		
PA5435	-	pyruvate carboxylase subunit B		0.282	
PA5436	-	acetyl-CoA carboxylase subunit A		0.400	
PA5440	-	peptidase		2.818	
PA5454	*rmd*	oxidoreductase Rmd	0.667		
PA5455	-	hypothetical protein	0.553		
PA5460	-	hypothetical protein			2.429
PA5462	-	hypothetical protein	0.514		
PA5472	-	hypothetical protein	0.695	2.400	
PA5479	*gltP*	glutamate/aspartate:proton symporter		2.362	
PA5481	-	hypothetical protein	6.688		6.226
PA5482	-	hypothetical protein	6.320		4.180
PA5491	-	cytochrome	0.530		
PA5495	*thrB*	homoserine kinase			2.141
PA5502	-	hypothetical protein	0.653		
PA5504	-	D-methionine ABC transporter	0.643		
PA5528	-	hypothetical protein	0.566		
PA5546	-	hypothetical protein		0.350	
PA5550	*glmR*	GlmR transcriptional regulator		5.951	
PA5553	*atpC*	ATP synthase F0F1 subunit epsilon		3.413	
PA5554	*atpD*	ATP synthase F0F1 subunit beta		2.543	
PA5555	*atpG*	ATP synthase F0F1 subunit gamma		2.692	
PA5556	*atpA*	ATP synthase F0F1 subunit alpha		2.843	
PA5557	*atpH*	ATP synthase F0F1 subunit delta		2.433	
PA5558	*atpF*	ATP synthase F0F1 subunit BATP synthase F0F1 subunit B		2.978	2.492
PA5560	*atpB*	ATP synthase F0F1 subunit A		2.321	

[a] Fold change of genes 30 min post-attachment on indicated surfaces compared to 5 min post-attachment on Ibidi µ-Slide, where a fold change of 1 indicates no difference. Green shading indicates genes with elevated RNA levels compared to 5 min (fold change > 1). Red shading indicates genes with reduced RNA levels compared to 5 min (fold change < 1). Grey shading indicates no sigicant difference in gene expression compared to 5 min

[b] Polycarbonate plastic

Table S3: Bacterial strains and plasmids used in this study

Strain or plasmid	Relevant genotype/characteristics	Ref or source
P. aeruginosa		
mPAO1	Parent PAO1 strain of PAO1 transposon library	(1)
mPAO1*pfpI::Tn*	Transposon mutant; PW1654; lacZwp04q1A07	(1)
mPAO1*phnA::Tn*	Transposon mutant; PW2808; lacZbp01q3D11	(1)
mPAO1*leuD::Tn*	Transposon mutant; PW6246; lacZwp05q2G10	(1)
mPAO1moaE::Tn	Transposon mutant; PW2470; phoAwp05q2C07	(1)
PAO1	Parent PAO1 strain of gene deletion mutants	
PAO1Δ*pslBCD*	Complete deletion of *pslBCD* genes. Mutant does not produce Psl	(2)
PAO1Δ*wspF*	Complete deletion of *wspF*. Mutant has elevated levels of c-di-GMP due to continued activation of WspR	(3)
Plasmids		
pUCP18	Empty vector for complementing constructs. Carb	
pUCP18::*pfpI*	Complementing vector. Wild type allele amplified from PAO1 as *Eco*RI and *HIn*dIII fragment. Carb	This study
pUCP18::*phnA*	Complementing vector. Wild type allele amplified from PAO1 as *Sac*I and *HIn*dIII fragment. Carb	This study
pUCP18::*leuD*	Complementing vector. Wild type allele amplified from PAO1 as *Sac*I and *HIn*dIII fragment. Carb	This study
pUCP18::*moaE*	Complementing vector. Wild type allele amplified from PAO1 as *Eco*RI and *HIn*dIII fragment. Carb	This study

Carb = carbenicillin at 300 µg/mL to maintain plasmid in *P. aeruginosa*

Table S4: Primers used in this study

Primer	Primer Sequence (5' - 3') [a]	R/E
*leuD*_F	CCCGAGCTCGACCGGGTATTCATCGGTTC	*Sac*I
*leuD*_R	CCCAAGCTTGGGTGGAATAGCGACTGAAGA	*Hin*dIII
*pfpI*_F	CCGGAATTCGAAACGGTTGAGGGTGACGA	*Eco*RI
*pfpI*_R	CCCAAGCTTGTTGATGCGATGAACCAGCA	*Hin*dIII
*moaE*_F	CCGGAATTCGCCCTGACCATCTACGACAT	*Eco*RI
*moaE*_R	CCCAAGCTTATGGTGGAAGTGCCGATCTC	*Hin*dIII
*phnA*_F	CCCGAGCTCGACTGAGACGGGACATCCAT	*Sac*I
phnA_R	CCCAAGCTTCAGCACCAGCAGTTCGCAA	*Hin*dIII

[a] Restriction enzyme site is underlined

References:

1. **Jacobs MA, Alwood A, Thaipisuttikul I, Spencer D, Haugen E, Ernst S, Will O, Kaul R, Raymond C and Levy R.** 2003. Comprehensive transposon mutant library of Pseudomonas aeruginosa. Proceedings of the National Academy of Sciences **100**:14339-14344.

2. **Kirisits MJ, Prost L, Starkey M and Parsek MR.** 2005. Characterization of colony morphology variants isolated from Pseudomonas aeruginosa biofilms. Appl Environ Microbiol **71**:4809-4821.

3. **Hickman JW, Tifrea DF and Harwood CS.** 2005. A chemosensory system that regulates biofilm formation through modulation of cyclic diguanylate levels. Proc Natl Acad Sci U S A **102**:14422-14427.
